# Guest Molecule-Mediated Energy Harvesting in a Conformationally Sensitive Peptide–Metal Organic Framework

**DOI:** 10.1021/jacs.1c11750

**Published:** 2022-01-24

**Authors:** Yu Chen, Sarah Guerin, Hui Yuan, Joseph O’Donnell, Bin Xue, Pierre-Andre Cazade, Ehtsham Ul Haq, Linda J. W. Shimon, Sigal Rencus-Lazar, Syed A. M. Tofail, Yi Cao, Damien Thompson, Rusen Yang, Ehud Gazit

**Affiliations:** † Department of Molecular Microbiology and Biotechnology, The Shmunis School of Biomedicine and Cancer Research, Tel Aviv University, Tel Aviv 6997801, Israel; ‡ Department of Physics, Bernal Institute, University of Limerick, Limerick V94 T9PX, Ireland; § School of Advanced Materials and Nanotechnology, Xidian University, Xi’an 710126, China; ∥ National Laboratory of Solid State Microstructure, Department of Physics, Nanjing University, Nanjing 210000, China; ⊥ Department of Chemical Research Support, Weizmann Institute of Science, Rehovot 7610001, Israel

## Abstract

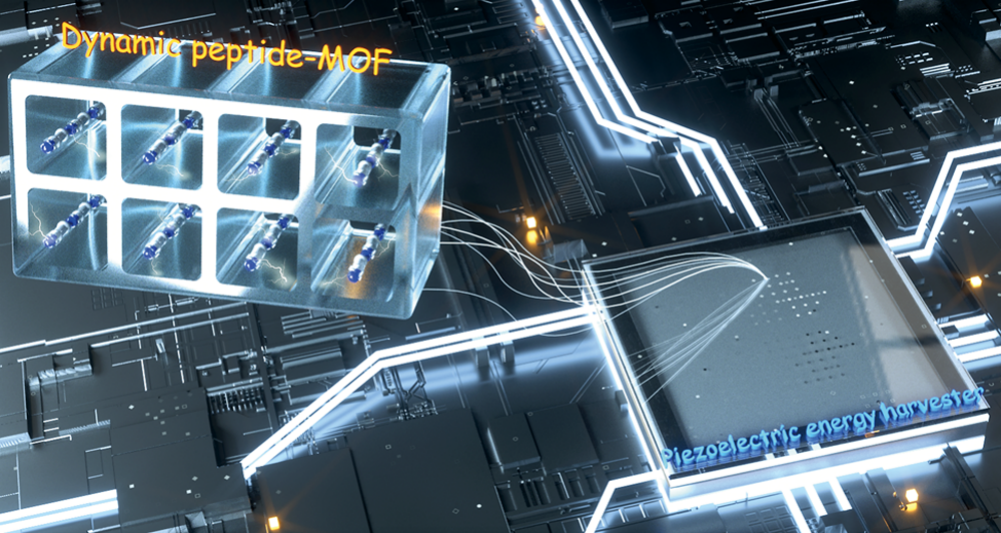

The apparent piezoelectricity of biological materials is not yet fully understood at the molecular level. In particular, dynamic noncovalent interactions, such as host–guest binding, are not included in the classical piezoelectric model, which limits the rational design of eco-friendly piezoelectric supramolecular materials. Here, inspired by the conformation-dependent mechanoresponse of the Piezo channel proteins, we show that guest–host interactions can amplify the electromechanical response of a conformationally mobile peptide metal–organic framework (MOF) based on the endogenous carnosine dipeptide, demonstrating a new type of adaptive piezoelectric supramolecular material. Density functional theory (DFT) predictions validated by piezoresponse force microscopy (PFM) measurements show that directional alignment of the guest molecules in the host carnosine–zinc peptide MOF channel determines the macroscopic electromechanical properties. We produce stable, robust 1.4 V open-circuit voltage under applied force of 25 N with a frequency of 0.1 Hz. Our findings demonstrate that the regulation of host–guest interactions could serve as an efficient method for engineering sustainable peptide-based power generators.

## Introduction

The conversion of mechanical force into cellular signals is a core biological function conserved throughout mammalian evolution,^1^ enabling essential biological functions including sense of touch,^2^ location and movement (proprioception),^3^ pain (nociception),^4^ and lung inflation.^5^ Previous studies have determined that the Piezo1 and Piezo2 proteins control their ion permeability properties through the conformational changes of the arranged piezo repeats channel induced by lateral membrane tension.^6^ The mechanically activated channel allows guest ions to pass through the cell membrane in response to mechanical stimuli, thereby imparting force sensitivity to cells and organisms.^7^ Although the exact mechanism nature of mechanotransduction in biological Piezo channels is still unknown, this conformation-dependent cation-selective mechanoresponse opens new horizons for designing high-performance, biocompatible and sustainable adaptive piezoelectric materials.

Peptide-based supramolecular materials have attracted growing attention due to their bio-inspired nature, ease of large-scale synthesis, and useful biodegradability.^8−26^ The carnosine (β-alanyl-l-histidine) dipeptide is an endogenous antioxidant found in the heart, skeletal muscle fibers, and brain.^27,28^ Carnosine has been shown to inhibit the oligomerization of Aβ-amyloid in rat brain endothelial cells,^29^ which may be due to its capability to form salt bridges with charged side chains and van der Waals contacts with core hydrophobic residues.^29,30^ In addition, carnosine is well-known to chelate divalent zinc cations, commonly termed polaprezinc, which is widely used in Zn supplementation therapy and for treating gastric ulcers.^31^

Implementing the design principles for the adaptive peptide-based metal–organic framework formulated by Rosseinsky and co-workers, scientists have synthesized topologically distinct two- or three-dimensional peptide-MOF architectures by changing the type and sequence of amino acids on peptide ligands.^32−39^ In particular, carnosine has been assembled into a three-dimensional chiral framework through the coordination of Zn(II).^33^ In the carnosine_Zn(II) (Car_Zn) peptide-MOFs, each carnosine linker connects to four Zn cations, and two of these cations are bridged with a deprotonated imidazole ring, forming a permanent microporous scaffold. Owing to the flexible alkyl segments in the β-alanine-histidine peptide, carnosine-based linkers can adopt a wide range of conformational states through low-energy torsional rearrangements, which enables guest-specific flexible response of the Car_Zn framework; that in turn affects the electromechanical behavior.^40^ Even though bio-inspired host–guest interactions have now been exploited in materials design,^41−43^ studies involving guest-modulated electromechanical behavior are relatively underexplored.^44,45^ The unambiguous demonstration of guest molecule-directed electromechanical properties would offer a means of dynamically modulating piezoelectric response, allowing better understanding of piezoelectricity in soft materials and providing an additional functionality for emerging eco-friendly piezoelectric devices.^46−51^

**Figure 1:**
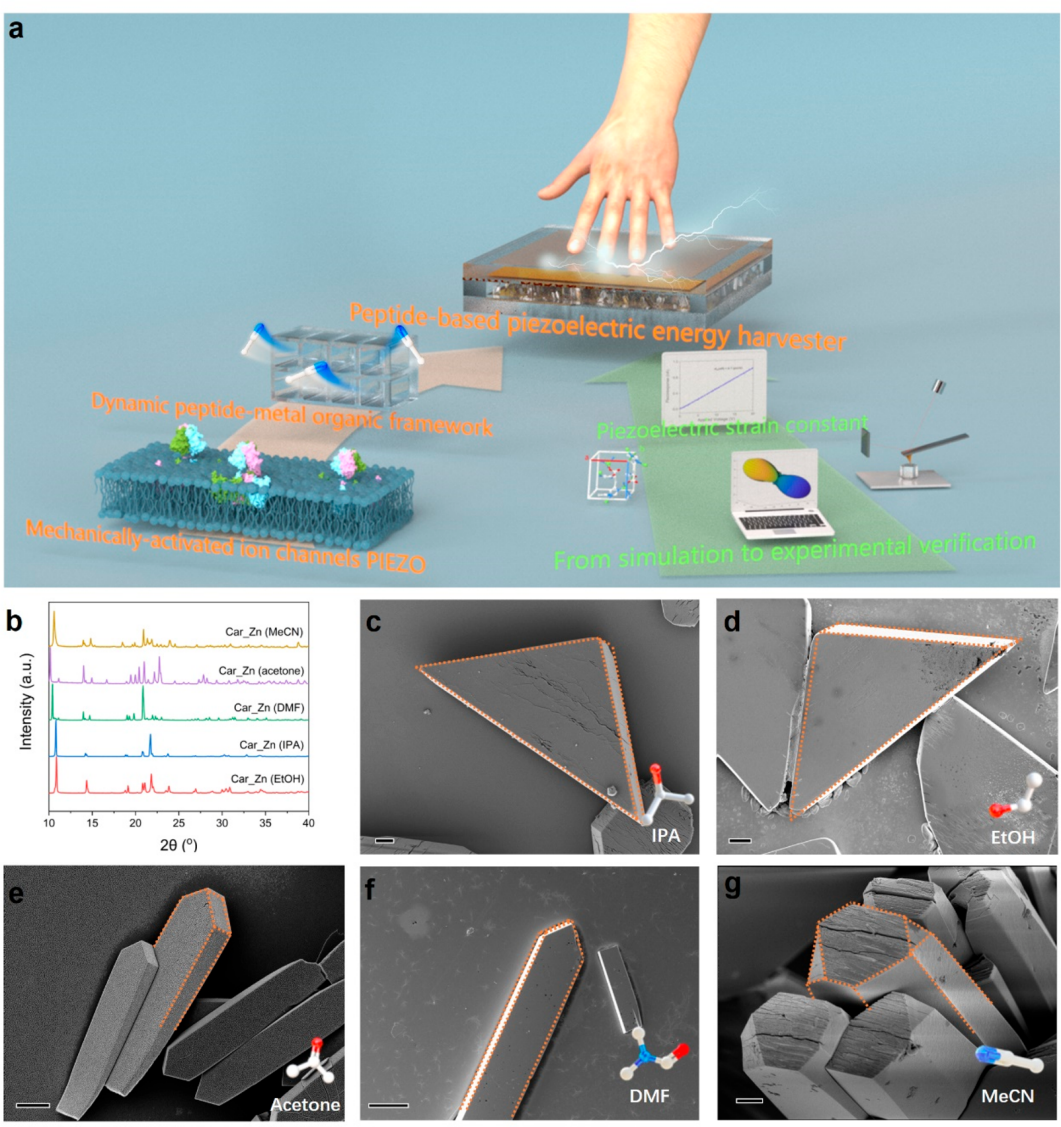
(a) Schematic illustration of the combination of methods used to decipher and optimize the guest molecule-mediated electromechanical properties of bio-inspired peptide-MOFs. Modeling-guided statistical PFM measures the piezoelectric tensor of peptide-MOF crystals, allowing us to map the relationship between microstructure and electromechanical behavior. (b) Powder X-ray diffraction (XRD) patterns of Car_Zn MOF incubated with five different guest molecules. (c–g) Scanning electron microscopy (SEM) images of neat prismatic morphologies of all assembled crystalline architectures (scale bar: 2, 3, 1, 10, and 3 μm, respectively), namely, the triangular prism morphology for Car_Zn with guest IPA or EtOH, rectangular prism morphology with acetone or DMF, and representative hexagonal prism morphology observed in Car_Zn·(MeCN).

Here, we report the large, guest-specific electromechanical response of Car_Zn peptide-MOFs assembled with five different guest molecules, namely isopropyl alcohol (IPA), dimethylformamide (DMF), acetone, acetonitrile (MeCN), and ethanol (EtOH). Atomic-level structural analysis of different peptide-MOFs obtained by X-ray crystallography revealed that the MeCN guest molecule uniquely directs assembly of a triclinic framework in which the polarization is not constrained by symmetry, which endows the crystal with 18 nonzero piezoelectric coefficients, including a measurable longitudinal *d*_33_ response. Density functional theory (DFT) predictive models were used to map the piezoelectric tensor of the full set of Car_Zn crystals and investigate the relationship between microstructure and electromechanical behavior (Figure 1a), validated by piezoresponse force microscopy (PFM). Our results showed that a significant piezoelectric response can be achieved by controlling the orientation of the guest molecules in the channel. As proof of concept, we demonstrated that the peptide-MOF with piezoresponsive morphology can provide the core active layer for a robust, stable energy-harvesting device with voltage outputs >1 V.

## Results and Discussion

Car_Zn MOFs were assembled via a zinc nitrate and carnosine reaction in the presence of five different guest molecules under controlled experimental conditions^33^ (see the Experimental Section in the Supporting Information). The powder X-ray diffraction (PXRD) patterns of the Car_Zn MOFs exhibited high crystallinity and were strongly consistent with the simulation data from the single crystal structure, signifying the same crystalline form (Figure 1b and Figures S1–S5). Scanning electron microscopy (SEM) characterizations demonstrated neat prismatic morphologies of all assembled crystalline architectures (Figure 1c–g). With the incorporation of IPA or EtOH (Figure 1c,d, Figures S6 and S7), Car_Zn MOFs exhibited an unusual triangular prism morphology measuring tens of micrometers in length, and the morphology could be clearly observed to become a rectangular prism when the guest was switched to DMF or acetone (Figure 1e,f, Figures S8 and S9). Because of the different solubility of the carnosine ligand in solvents of different polarity, the nucleation and growth of Car_Zn MOF could be significantly affected by the choice of solvent^52^ (Figure S10). As suggested by the morphological analysis of the Car_Zn·(EtOH) single crystal (Figure S11), the anisotropic growth of the (020) surface along the [001] direction was suppressed mainly by the addition of polar protic alcohol, which could form a shell of hydrogen bonded alcohol molecules around the carnosine linker.^33^ Furthermore, because the shape of a triangular prism can be approximated as a truncation of a rectangular prism, in terms of morphology, these solvent molecules provide similar crystalline habits for the growth of Car_Zn MOFs. Intriguingly, the addition of MeCN induced a new structure polymorph with a hexagonal prism crystal morphology, with a length up to 60 μm (Figure 1g and Figure S12). This unique morphology change may be attributed to the solvent template effect with the induced conformational distortion of the assembly triggering a phase transition to create the electromechanically active framework.^52−55^

**Figure 2:**
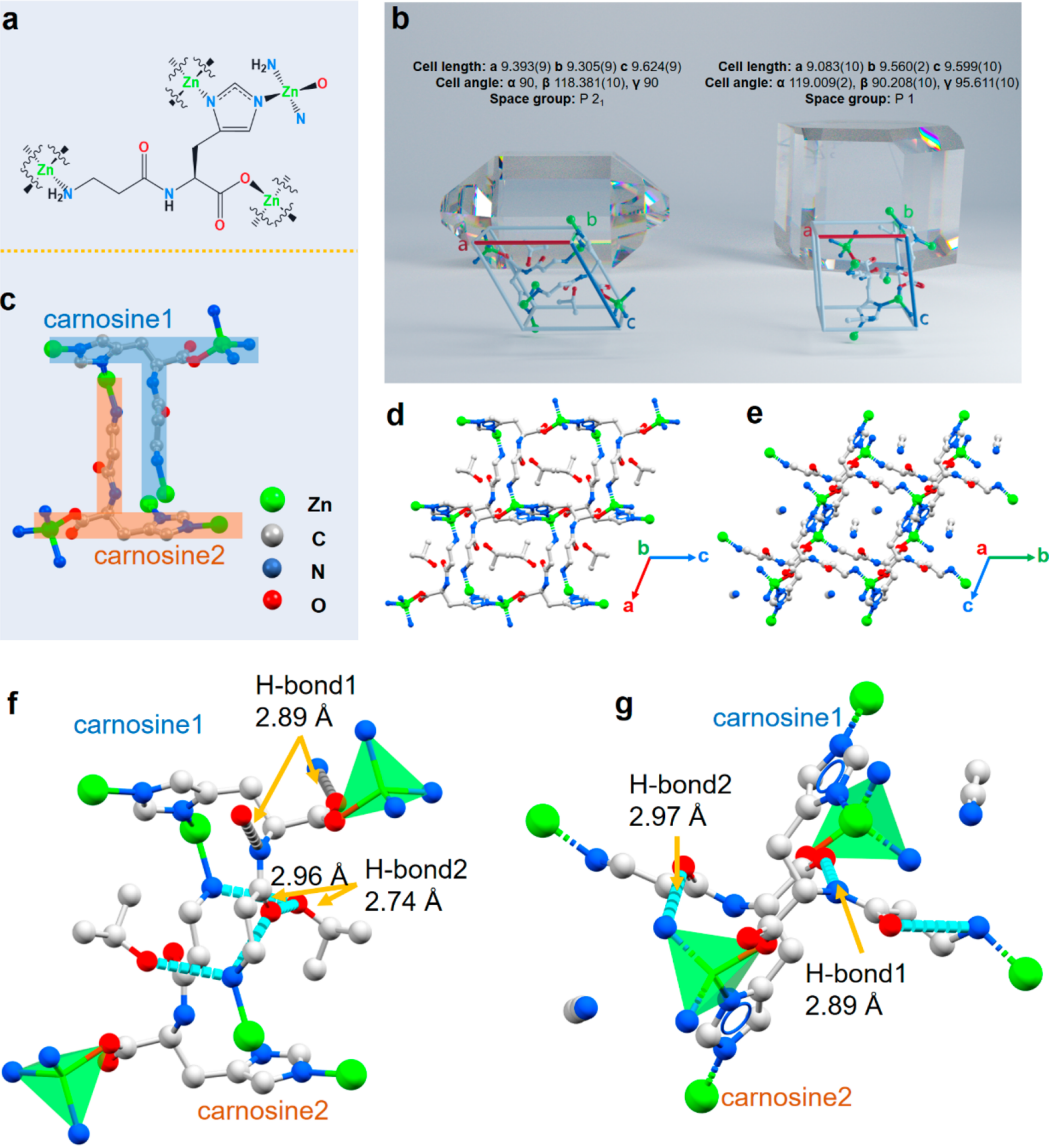
Structural analysis of Car_Zn MOFs. (a) The carnosine molecule links four tetrahedral Zn cations. (b) The unit cell parameters of Car_Zn·(IPA) (left panel) and Car_Zn·(MeCN) (right panel) and their prismatic morphologies predicted by the Bravais, Friedel, Donnay, and Harker (BFDH) method. (c) Reverse-interdigitated arrangement of the “T” shape of carnosine facilitates the formation of lozenge-shaped channels. Color code: green, Zn; gray, C; blue, N; and red, O. (d, e) View down the one-dimensional channels of (d) Car_Zn·(IPA) and (e) Car_Zn·(MeCN) shows the change in channel shape and orientation caused by the guest molecule. (f, g) Hydrogen-bonding pattern of (f) Car_Zn·(IPA) and (g) Car_Zn·(MeCN) illustrates how the nature of the guest molecule in the channel affects the conformation of the peptide linker through hydrogen bonds.

To further characterize the specific guest molecule-mediated assembly mechanism at the molecular level, the as-prepared crystals were thoroughly analyzed via X-ray crystallography (Figures S13–S16 and Table S1). Under the above-mentioned different solvent conditions, the imidazole and carboxylic acid groups of carnosine were deprotonated to coordinate with Zn(II), forming the Car_Zn framework (Figure 2a). The Zn(II) ion was coordinated with one carboxy- and one amino-terminal group on carnosine molecules and two nitrogen atoms of the imidazole group, providing a geometric tetrahedron with a Zn(II) central coordination site. As illustrated by the representative Car_Zn·(IPA) unit cell (left panel of Figure 2b), the Car_Zn complex crystallized in the monoclinic space group *P*2_1_ with one guest molecule and one neutral [Zn(L)] complex per asymmetric unit. However, in the Car_Zn·(MeCN) polymorph (right panel of Figure 2b), the carnosine linker assumed two conformations that were no longer symmetry-equivalent, unlike the other Car_Zn analogues. The two independent conformations of carnosine are present in the asymmetric unit formed with MeCN, yielding significantly altered unit-cell parameters and thereby lowering the symmetry of the unit cell from monoclinic to triclinic *P*1. The preferred protonation states and bonding patterns described above were confirmed through extensive DFT calculations of alternative molecular states in the XRD unit cells. The measured Zn–carnosine complexation sites (Figure 2a) clearly show that the imidazole nitrogen atoms are both unprotonated and that the terminal groups are amino NH_2_ and carboxylate COO^–^. The favorability of the amide state RC(=O)–NHR′ in the chain was confirmed by comparing with the alternative iminol RC(OH)=NR′,^56^ which did not fit the observed carnosine–Zn crystal packing with coordinated ordered solvent molecules.

Going beyond the crystal unit cell and examining longer-range superstructure motifs, the reverse-interdigitated arrangement of the “T”-shaped carnosine (Figure 2c) allowed the formation of lozenge-shaped channels filled with guest molecules and aligned along the *b*-axis (Figure 2d and Figures S17–S20). Although the solvent-filled channels could still be observed in Car_Zn·(MeCN), the alignment of the pores changed from the *b*-axis to the *a*-axis (Figure 2e and Figure S20). In addition, the torsional flexibility of the carnosine ligand allowed the Car_Zn framework to be structurally sensitive to the template effect of the specific guest molecules. In each structure, the carnosine linker achieved a distinct conformation by adjusting its torsion angles, responding to the size, shape, and hydrogen-bonding characteristics of the guest molecules in the Car_Zn framework channel (Figure 2f,g). During assembly with IPA guest molecules, the carnosine linker twisted at φ1 = 178.45° and φ2 = −64.18° (Figure S21), orienting the carnosine amide and amine groups toward the IPA, forming guest–host (IPA–Car) hydrogen bonds (O4–H4A···O1, 2.74 Å; N1–H1A···O4, 2.96 Å) (Figure S22). Those guest–host hydrogen bonds together with the host–host (Car–Car) intermolecular hydrogen bonds (N2–H20···O3, 2.89 Å) and carnosine–Zn(II) coordination bonds stabilized the framework (Figure 2f). However, upon switching to MeCN guest molecules, the amide groups on the carnosine stacked along the channel wall to form an antiparallel β-sheet-like hydrogen bond network (N2–H2···O6, 2.89 Å; N6–H6···O3, 2.97 Å) between the carboxylate and amine group on the adjacent linker (Figure S23). Uniquely with MeCN, the guest molecule did not form hydrogen bond interactions with the carnosine molecules, and the Car_Zn·(MeCN) framework was solely stabilized through the carnosine–Zn(II) coordination bonds and host–host intermolecular hydrogen bonds (Figure 2g). From a crystal engineering perspective, it can be inferred that the solvent used here not only served as a bulk reaction medium but also acted as a structure-directing agent, embedding in the framework through guest–host interactions to profoundly affect the morphology and atomistic packing structure of the final Car_Zn MOF.

The noncentrosymmetric structure with the directional polar guest molecule array and hydrogen-bonding networks signifies internal polarization, implying piezoelectric properties.^57,58^ We used DFT calculations to predict the elastic, dielectric, and piezoelectric constants of the Car–Zn MOFs. Full details of the computational methodology can be found in the Methods section. The crystals fell into three distinct mechanical regimes according to their computed elastic properties (Tables S2 and S3, Figures S24–S27), with the ethanol guest solvent molecule producing the stiffest crystal with a predicted Young’s modulus of 27 GPa. With the incorporation of acetone or DMF, the predicted Young’s modulus of the crystals was reduced to ∼14–17 GPa, while MeCN or IPA solvents produced significantly weaker crystals with a predicted Young’s modulus of 7–9 GPa. The Car_Zn MOF containing guest molecules of EtOH, DMF, and acetone then showed the lowest piezoelectric strain constant (Tables S4–S8), reflecting their increased average elastic stiffness constants and lower piezoelectric polarization. DMF, acetone, and EtOH guests produce MOFs with predicted *d*_16_ = 6.9, 11.2, and 5.5 pC/N, respectively.

Despite their differing mechanical properties, both alcohol-containing crystals Car_Zn·(EtOH) and Car_Zn·(IPA) showed a nearly identical range of piezoelectric strain constants with maximum values of 7.3 and 5.5 pC/N, respectively (Tables S7 and S8), showing that increased flexibility in the IPA crystal balances its decrease in charge tensor values. Furthermore, as we revealed in the single crystal diffraction results, Car_Zn·(MeCN) crystallized with significantly altered unit cell parameters and adopting a lower symmetry unit cell (monoclinic to triclinic), thus allowing for a complete 18 nonzero component piezoelectric tensor (Figure S28). While its predicted *d*_33_ value of 4.7 pC/N is modest, it has six piezoelectric strain constants between 9.8 and 18.2 pC/N (*d*_max_ = *d*_15_). In common with most biological materials,^46,59^ the low dielectric constants of the peptide-MOF structures permit significant voltage constant outputs, here as high as 435 mV m/N (*g*_15_) (Table S4). These values can be attributed to an overall lower elastic stiffness and |*e*_*ij*_| values between 0.15 and 0.33 C/m^2^.

**Figure 3:**
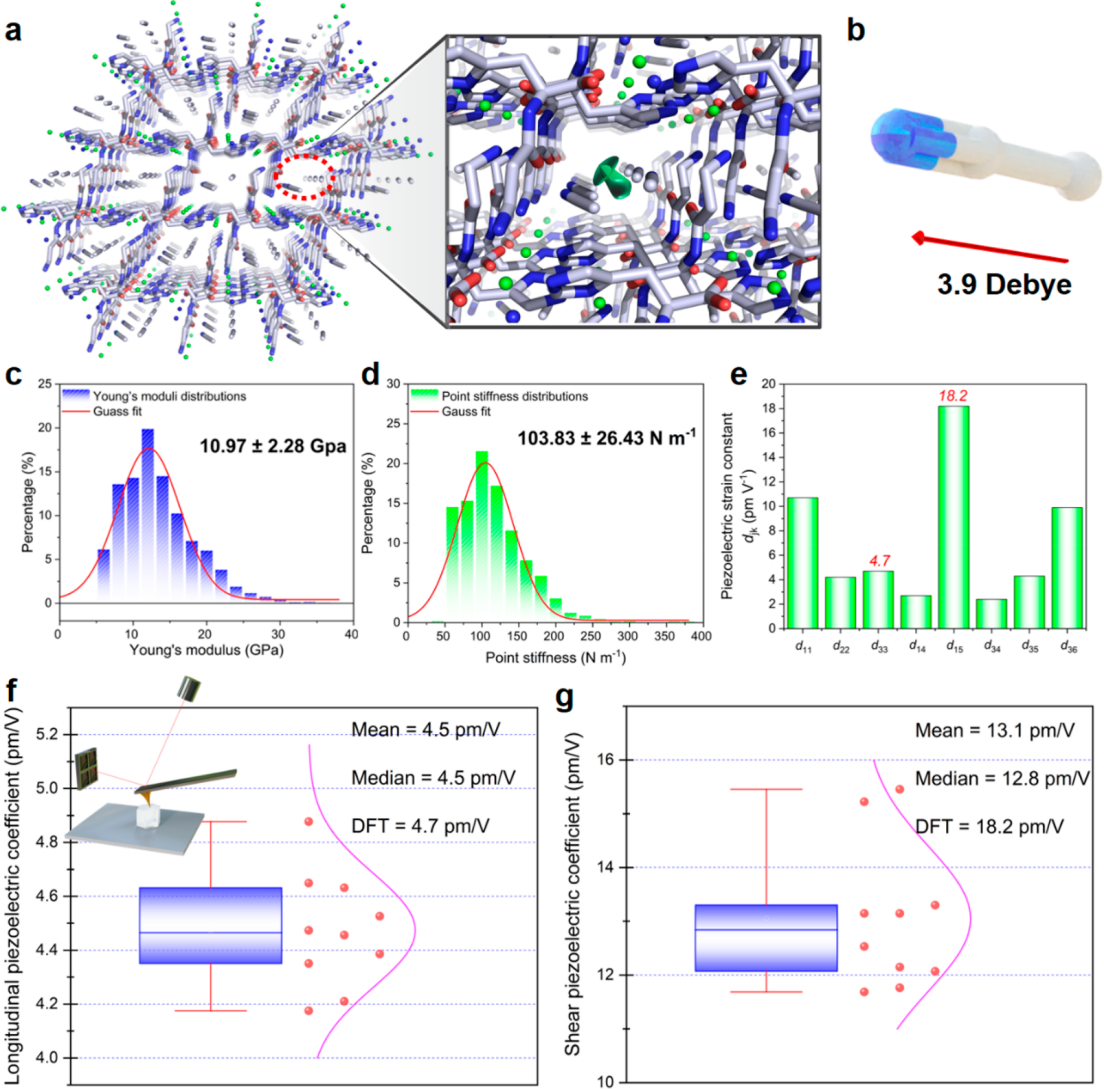
Mechanical and piezoelectric properties of the Car_Zn·(MeCN) crystal. (a) Directional guest solvent MeCN molecule alignment (circled in red) in the Car_Zn framework channel and molecular dipole sum to a spontaneous crystal polarization (green arrow in right panel) along the *a*-axis. (b) Molecule dipole of MeCN. (c) Young’s modulus and (d) point stiffness statistical distributions of the Car_Zn·(MeCN) crystal. (e) Calculated piezoelectric strain constants for the Car_Zn·(MeCN) crystal. (f, g) Experimental measurement of piezoelectric coefficients using PFM. (f) Statistical distribution of the vertical *d*_L_^eff^ coefficients. (g) Statistical distribution of the shear *d*_S_^eff^ coefficients. The mean and median values for each distribution are shown alongside the theoretical maximum DFT value to demonstrate the good correspondence between DFT predictions and experimental measurements.

The computed electromechanical properties stem from the guest molecule-mediated carnosine packing patterns in the crystals. For Car_Zn·(MeCN), the DFT models predict decrease in the largest elastic stiffness constant (and hence the Young’s modulus, Tables S2 and S3), primarily due to the directional alignment of the small solvent molecules (circled in red, Figure 3a) that introduced more flexibility in the packing of the carnosine chains. There was also less variation in stiffness along each axis (Table S2), as the carnosine molecules were equally bisected along the *b*- and *c*-axes (Figure 3a). The Zn ions and solvent molecules bridged the largest inter-carnosine gaps along the *a*-axis, maintaining the mechanical strength and amplifying the polarization. The maximum piezoelectric polarization (−0.33 C/m^2^, Table S4) was found along the acetonitrile MeCN solvent molecular dipole^60^ (3.9 D, Figure 3b), which aligned with the *a*-axis as indicated by the green arrow in the right panel of Figure 3a. The maximum predicted axial polarization (0.3 C/m^2^ along *a*, 0.1 C/m^2^ along *b*, and 0.2 C/m^2^ along *c*) was inversely proportional to the interion spacing along each axis (6 Å along *a*, 10 Å along *b*, and 8 Å along *c*), which demonstrates how guest molecule-directed supramolecular packing determines the electromechanical response by stabilizing specific interion spacing. Thus, the most significant piezoelectric charge constants in the Car_Zn·(MeCN) crystal were along the *a*-axis (Table S4). On the other hand, in the Car_Zn·(IPA) crystal (Figure S27), the channels were off-center near the corners of the cell, so when a shearing force was applied along the *c*-axis, it was much easier to push the molecular layers close together (*c*_66_ = 2.8 GPa, the lowest stiffness constant of all the crystals), enabling a maximum *d*_36_ piezoelectric response of 7.3 pC/N (Table S8). The relatively low charge tensor values in the crystals containing alcohol guest molecules may be due to the juxtaposed orientations of the two alcohol molecules in each channel, resulting in the alcohol molecular dipoles canceling out rather than contributing to the piezoelectric response. As discussed above, the orientation of the guest molecules in the channel controls the macroscopic electromechanical properties of the MOFs, with the highest piezoelectric responses obtainable from the favorable alignment of the crystallized guest molecules (Table S9).

To experimentally validate the DFT predicted stiffness tenors, atomic force microscopy-based nanoindentation experiments were employed to investigate the mechanical properties. The measured elasticity of the Car_Zn·(MeCN) crystal showed a Young’s modulus of 10.97 ± 2.28 GPa along the thickness direction, which led to a point stiffness of 103.83 ± 26.43 N m^–1^ of the crystal (Figure 3c,d). This value is consistent with the DFT calculation results and confirms that the peptide-based porous host has a conformational energy landscape similar to that of flexible macromolecules but still retains the rigidity conferred by the Zn–imidazole coordination bond.^33,34^ Furthermore, piezoresponse force microscopy (PFM) was applied to investigate the piezoelectricity of the Car_Zn·(MeCN) crystal. During the PFM measurement of the piezoelectric response, the tip of the PFM was in contact with the single crystal and remained stationary during the entire measurement process. The piezoelectric response of the single crystal was extrapolated from the linear relationship between the applied voltage (in volts) and the resulting deformation (in picometers).^61^ For the out-of-plane response, the resulting deformation was proportional to the effective longitudinal piezoelectric coefficient, which can be denoted as *d*_L_^eff^ (Figure S29a). Likewise, for the in-plane response, the resulting deformation was proportional to the effective shear piezoelectric coefficient (*d*_S_^eff^) (Figure S29b). The linear relationship between the vertical and in-plane piezoelectric response as measured by the photodiode system and applied voltage indicated a genuine piezoelectric property of the Car_Zn·(MeCN) crystal (Figures S30–S32). The results reveal that the largest measured *d*_L_^eff^ of Car_Zn·(MeCN) is 4.7 pm/V and the largest *d*_S_^eff^ is 15.5 pm/V, in very good agreement with the theoretical maximum values predicted by DFT of 4.7 and 18.2 pm/V, respectively (Figure 3e–g).

**Figure 4:**
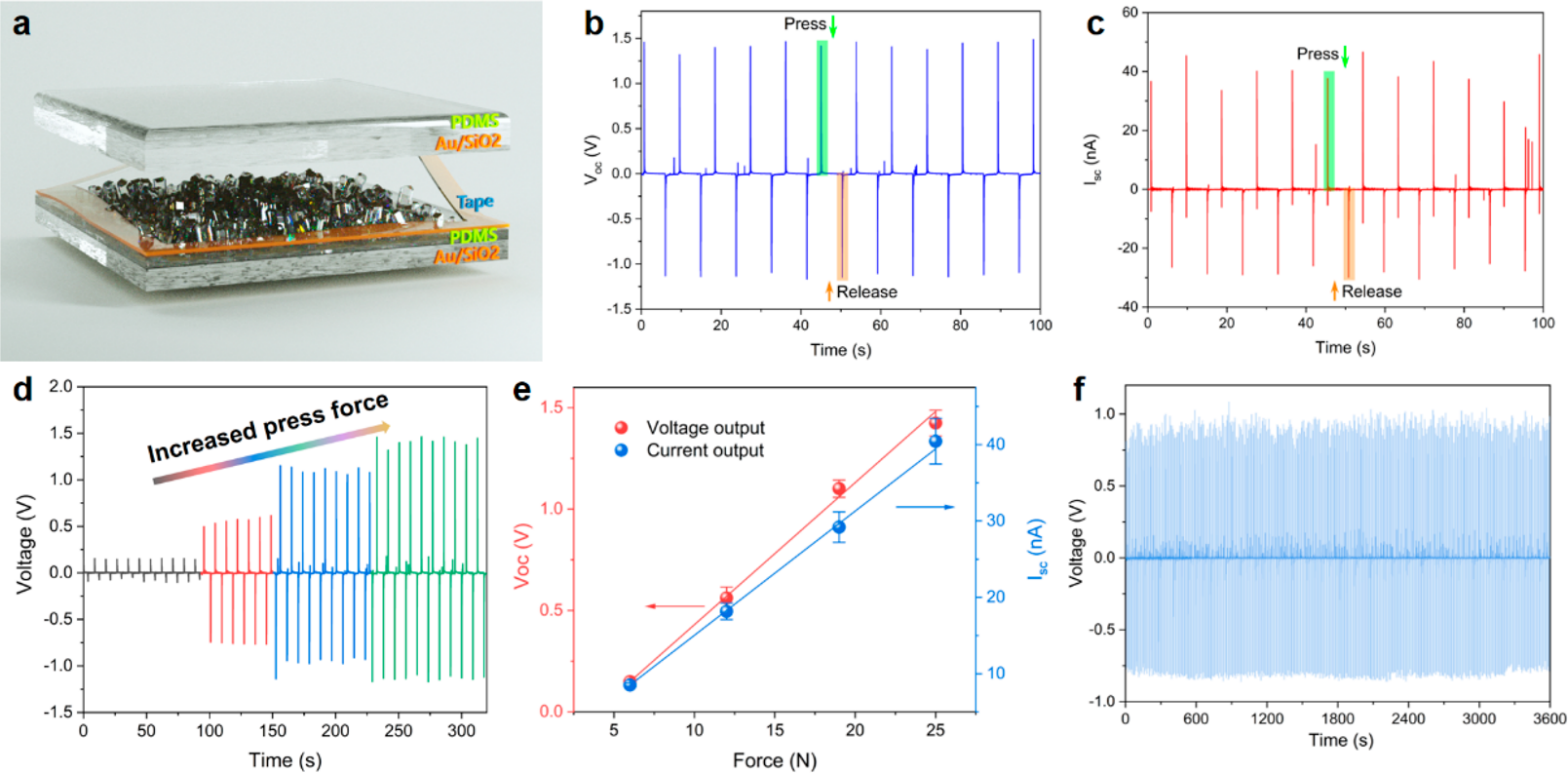
Characterization of the peptide-MOF-based generator. (a) Schematic configuration of the generator using the Car_Zn·(MeCN) crystal as the active component. (b) Open-circuit voltage and (c) short-circuit current obtained from the generator with an applied periodic compressive force of 25 N. (d) Open-circuit voltage and (e) short-circuit current of the generator as a function of the applied force. (f) Stability measurement of the peptide-MOF-based generator. The open-circuit voltage was recorded continually under ∼19 N applied force at a frequency of 0.1 Hz.

Intrigued by the piezoelectric properties of the characterized peptide-MOF Car_Zn·(MeCN) crystal, we tested the potential of the peptide-MOF crystals for use as the active layer in a prototype piezoelectric energy generator.^62,63^ A coin-sized power generator was designed and fabricated by tightly sandwiching the Car_Zn·(MeCN) crystals array between two gold-coated silicon dioxide substrates connected to an external measuring instrument (Figure 4a). The entire device was firmly laminated with Kapton tape to prevent mechanical stress, dust, and moisture damage^64^ (Figure S33). A constant and stable mechanical force was applied to the generator through the dynamic mechanical test system, and the short-circuit current and open-circuit voltage were measured to characterize the generated electrical output signal. The peptide-MOF-based device-generated the corresponding periodic current (40.45 ± 2.99 nA) and voltage (1.42 ± 0.063 V) output signals when the generator was periodically compressed with 25 N force (Figure 4b,c). Furthermore, the obtained linear relationship with a slope of 1.62 nA/N and 0.07 V/N between applied force and current and voltage output values, respectively, demonstrated the linear piezoelectric response of the Car_Zn·(MeCN) crystal (Figure 4d,e and Figure S34). Finally, the stability measurement suggested that power generation could be sustained under a cyclic force (19 N). The output voltage showed no attenuation over 400 press/release cycles for an hour (Figure 4f and Figure S35), indicating the high durability of the peptide-based devices.

## Conclusions

In this study, we developed a simple but powerful “guest molecule-mediation effect” approach for tailor-made design of piezoelectric peptide-MOF crystals. We show the guest molecule MeCN selectively alters the crystal morphology and acts as a structure-directing agent to lower the symmetry of the unit cell. As a result, unlike the four other guests we tested, the Car_Zn·(MeCN) MOF crystallized into the lowest symmetric system (space group *P*1) with unconstrained polarization, which created a significant piezoresponse as mapped by using DFT calculations and microscopy. Reminiscent of transmembrane proteins and diphenylalanine peptide nanotubes, the extensive directionally aligned guest molecules in the narrow channel form a macroscopic dipole that can couple with shear force to generate the piezoelectric response. As a proof of concept, we demonstrate the utilization of a stable, robust peptide-MOF with useful 1 V output in a prototype sustainable, eco-friendly power generator. Our findings illustrate the rational modulation of peptide-MOFs to embed tailored functionalities and pave the way for supramolecular engineering of piezoelectric biomaterials for nanotechnology applications through further manipulation and design of internal host–guest interactions that confer dramatic changes in materials morphology and improve device performance.

## Supporting Information Available

Experimental details, materials, methods, and additional figures and tables (PDF)

## Accession Codes

CCDC 2104697–2104700 contain the supplementary crystallographic data for this paper. These data can be obtained free of charge via www.ccdc.cam.ac.uk/data_request/cif, or by emailing data_request@ccdc.cam.ac.uk, or by contacting The Cambridge Crystallographic Data Centre, 12 Union Road, Cambridge CB2 1EZ, UK; fax: +44 1223 336033.
