# Supplementary material for: Guest Molecule-Mediated Energy Harvesting in a Conformationally Sensitive Peptide–Metal Organic Framework
Source: J Am Chem Soc. 2022 Jan 24;144(8):3468–76. doi: 10.1021/jacs.1c11750 (PMC8895394; doi:10.1021/jacs.1c11750)
Supplement: Supplementary file 1 — ja1c11750_si_001.pdf [file ja1c11750_si_001.pdf]

Supplementary information

for

Guest molecule-mediated energy harvesting in a conformationally sensitive peptide-metal organic framework

Yu Chen [a†], Sarah Guerin [b†], Hui Yuan [c†], Joseph O'Donnell [b], Bin Xue [d], Pierre-Andre Cazade [b], Ehtsham Ul Haq [b], Linda J. W. Shimon [e], Sigal Rencus-Lazar [a], Syed A. M. Tofail [b], Yi Cao [d], Damien Thompson [b*], Rusen Yang [c*], Ehud Gazit [a*]

[a] Department of Molecular Microbiology and Biotechnology, The Shmunis School of Biomedicine and Cancer Research, Tel Aviv University, Tel Aviv, 6997801, Israel.

[b] Department of Physics, Bernal Institute, University of Limerick, Limerick, V94 T9PX, Ireland.

[c] School of Advanced Materials and Nanotechnology, Xidian University, Xi'an, 710126, China.

[d] National Laboratory of Solid State Microstructure, Department of Physics, Nanjing University, Nanjing, 210000, China.

[e] Department of Chemical Research Support, Weizmann Institute of Science, Rehovot, 7610001, Israel.

E-mail: ehudga@tauex.tau.ac.il

E-mail: Damien.Thompson@ul.ie

E-mail: rsyang@xidian.edu.cn

Table of Contents

Experimental Section and Materials..3

1. Materials 3

2. Crystal preparation..3

3. Characterization 3

Supplementary Figures..7

Supplementary Tables 42

References 51

Experimental Section and Materials

1. Materials

L-Carnosine (β-Ala-His-OH) with purity of 99.55% was purchased from Chem-Impex International, Inc. Zinc nitrate ($Zn(NO_3)_2$), dimethylformamide (DMF), ethanol (EtOH), isopropanol (IPA), and acetonitrile (MeCN) were purchased from Sigma-Aldrich (Rehovot, Israel). All materials were used as received without further purification. Water for all the experiments was processed using a Millipore purification system (Darmstadt, Germany) with minimum resistivity of 18.2 MΩ cm.

2. Crystal preparation.

The Car_Zn MOF crystals in different solvent were prepared following literature protocol[1]. In a typical synthesis procedure, the weighed carnosine powder was dissolved in water, to a concentration of 80 mM. A stock solution of carnosine (2 mL, 80 mM), $Zn(NO_3)_2$ (1 mL, 160 mM), solvent (4 mL, DMF, IPA, EtOH, Acetone or MeCN), and water (2 mL) were mixed in a 20 mL scintillation vial under vigorous sonication. The vial was heated at 100 °C for 4 h with ramping rate of 1 °C /min and was cooled down to room temperature at 1 °C /min. The resulting crystal was washed several times with ethanol and deionized water, then dried at 60 °C for 12 hours.

3. Characterization

Scanning electron microscopy (SEM). The silicon substrate was ultrasonically cleaned with acetone followed by isopropanol, and water for 5 minutes. Then, 10 µL Car_Zn MOF crystal samples were drop-casted on a silicon substrate and heated for 10 minutes. The Quorum SC7620 sputter coater system was used to sputter gold on the crystal samples at 15 mA for 45 seconds to improve the conductivity of the sample surface. SEM images were collected using Zeiss Gemini 300 (Zeiss, Germany) with an operating voltage of 3 kV. Images were collected in Inlens and secondary electron mode by using an Everhart-Thornley detector, with probe current of 237 pA and system vacuum lower than 9×10^{-7} mbar.

X-ray crystallography. Crystals suitable for diffraction were coated with Paratone oil (Hampton Research), mounted on loops and flash frozen in liquid nitrogen. Single crystal X-ray diffraction data measurements for Car-Zn·(IPA), Car-Zn·(EtOH), and Car-Zn·(acetone) were performed using a Bruker Kappa ApexII system with MoKα radiation (λ=0.71073Å). Data were collected and processed using the Apex3 suite of programs (Bruker 2018). Single crystal X-ray diffraction data measurements for Car-Zn·(MeCN) were performed using a Rigaku XtaLabPro system with MoKα radiation (λ=0.71073Å). Data were collected and processed using the CrysAlisPro suite of programs (RigakuOD 2018). The structures were solved by direct methods using SHELXT-2016/4 and refined by full-matrix least squares against F2 with SHELXL-2016.

Powder X-ray diffraction (PXRD). Car_Zn MOF crystal powder samples were deposited on a quartz zero-background sample holder. The diffraction patterns were collected using a D8ADVANCE diffractometer (Bruker, Germany) equipped with a linear detector LYNXEYE XE. Data collection was performed at room temperature with a scan range 2θ of 10–40°.

Optical microscopy. The Car_Zn·(EtOH) single crystals casted on a glass slide were directly observed using a Nikon Eclipse Ti-E fluorescence microscope at bright field channels.

Density functional theory (DFT) calculations. DFT electromechanical properties were predicted from periodic DFT[2] calculations on the single crystals using the VASP[3] code. Electronic structures were calculated using the PBE functional[4] with Grimme-D3 dispersion corrections[5] and projector augmented wave (PAW) pseudopotentials[6]. The crystal structure was optimised using a plane wave cut-off of 600 eV with a 4x4x4 k-point grid. A finite differences method was used to calculate the stiffness tensor, with each atom being displaced in each direction by ± 0.01 Å, and piezoelectric strain constants and dielectric tensors were calculated using Density Functional Perturbation Theory[7] (DFPT), with a plane wave cut-off of 1000 eV and k-point sampling of 2x2x2. Young's moduli were derived from the stiffness and its inverse compliance matrix components. Values are presented as an average of three calculation methods: the orthorhombic and triclinic approximations of Nye[8], and the Voigt-Reuss-Hill method[9,10]. All three methods give reasonable match with known experimental values for organic and inorganic crystals[11–13], so an arithmetic mean over the three analysis methods was used. Crystal structures were visualised using VESTA[14]. Using the piezoelectric charge coefficients, e_{ij}, calculated directly by VASP, and the elastic stiffness constants, c_{kj}, we could calculate the more useful piezoelectric strain coefficient, d_{ik}, using the relationship

$$d_{ik} = {e_{ij}}/{c_{kj}}$$

To obtain the voltage constants g_{ik}, we divided the corresponding piezoelectric strain constant $d_{ik,}$ by the relevant dielectric constant ε_{ii}. These constants are measured in Vm/ N.

$$g_{ik} = {d_{ik}}/{\varepsilon_{ii}\ \varepsilon_o}$$

AFM nano-indentation experiments.

Atomic force microscopy (AFM) nano-indentation experiment was carried out using a commercial AFM (JPK, Nanowizard IV, Berlin, Germany). The force curves were obtained and analysed using the commercial software from JPK. Silica cantilevers (RTESPA-525, Bruker Company with the half-open angle of the pyramidal face of θ: < 10°, tip radius: ~5 nm, spring constant: ~200 N m^{-1}) and QI mode (conditions: pixels:20× 20; Z length: 0.3 μm; extend and retract speed: 30 μm s^{-1}; Z resolution: 80000 Hz; maximum loading force: 800 nN) were used in all experiments. During the experiments, the peptide-MOF crystals were drop-casted on the surface of the quartz substrate, and the cantilever was moved above the crystal. The cantilever was brought to the crystal samples at a constant speed of 30 μm s^{-1} and held on the crystal surface at a constant force of 800 nN. The force-displacement curves were recorded. Young's modulus of the crystals could be obtained by fitting the extended curve with the Hertz mode,

$$F = \frac{4}{3}\frac{E}{(1-v^2)}\sqrt{R}\delta^{3/2} \quad (1)$$

F corresponds to the force, δ corresponds to the depth of the crystal pressed by the cantilever tip, R corresponds to the radius of the tip, E is the Young's modulus of the crystals and ν is the Poisson ratio (ν = 0.3). The point stiffness was determined as the normal force divided by the deformation of the sample and calculated from the force-displacement curves after deducting the deformation of the cantilever. For each sample, more than 5 regions were randomly selected to perform the experiment and the data collected using the JPK software. At least three cantilevers were used in the experiments to exclude the tip-to-tip dependency.

Piezoelectric characterization.

Piezoresponse force microscopy (PFM) was measured using an NT-MDT Ntegra Spectra operating in contact PFM mode[15–17]. Generally, in this mode, the atomic force microscopy probe is in contact with the sample, and an AC voltage is applied to generate a piezoelectric response within the sample which is then measured. The PFM detects crystal surface vibrations caused by the action of an external electric field due to the converse piezoelectric effect. The PFM system sweeps the voltage between 0 and 50 V and back again and simultaneously measure the magnitude of the piezoresponse detected by the photodiode system. For the PFM experiment of the Car_Zn·(MeCN) crystal, two sites along the crystal were selected. At each site of the crystal, five measurements were performed in an area of 2 μm^2. The effective longitudinal piezoelectric coefficient (d_L^{eff}) of the sample is proportional to the slope of the piezoresponse curve and then can be calculated using the following equation:

$$d_{33}^{eff}\left(\frac{pm}{V}\right) = \frac{Slope\ of\ piezoresponse\ curve\left(\frac{nA}{V}\right)}{Gain \times Input \times IOS\left(\frac{nA}{nm}\right)} \times 1000$$

The inverse optical sensitivity (IOS) coefficient is a conversion factor, which is related to the unit of deformation recorded by the photodiode to the unit needed for quantification. It depends on the alignment of the laser on the tip and the reflectivity of the back surface of the tip. The vertical IOS (IOS_V) is calculated from the slope of a force-distance curve performed on a hard substrate. In this case, the IOS_V was measured to be 0.057 nA/nm. The Gain and Input are both experimental factors selected during scanning and in this case were both equal to 10. According to the linear relationship between the vertical piezoelectric response of the Car_Zn·(MeCN) crystal and the applied voltage measured by the photodiode system (**Supplementary Figure S28**), the slope of the piezoelectric response curve could be fitted to 0.0265 nA/V. Therefore, evaluation of the d_L^{eff} piezoelectric coefficient of the Car_Zn·(MeCN) crystal yielded d_L^{eff} = 4.65 pm/V. To calculate the effective shear piezoelectric coefficient (d_S^{eff}), the lateral inverse optical sensitivity should be determined. A simple geometric relationship between cantilever length (L), tip height (h), and the ratio of

vertical sensitivity to lateral sensitivity (R) can be used to determine the lateral IOS (IOS_L) using the equation:

$$R = \frac{IOS_V}{IOS_L} = \frac{2L}{3h}$$

IOS_L can then be used to determine the d_S^{eff} coefficient. Accordingly, the piezoelectric coefficient d_S^{eff} of the Car_Zn·(MeCN) crystal could be determined to be 15.53 pm/V (**Supplementary Figure S28**). To ensure the quantification method accuracy, both positive (**Supplementary Figure S29**, periodically poled lithium niobate (NT-MDT, Russia), Y-cut LN (Siegert Wafer, Germany)) and negative (**Supplementary Figure S30**, amorphous glass) control samples were used.

Fabrication of peptide-MOF-based generator.

In a typical sandwich-type peptide-MOF-based generator fabrication process, an Au-coated SiO_2 substrate with a size of 1×1 cm^{-2} was used as the top and bottom electrodes. The Au/SiO_2 substrate was placed at the bottom of the scintillation vial during the Car_Zn·(MeCN) crystal assembly process, allowing the crystal to grow on the surface of the gold layer. After the Car_Zn·(MeCN) crystal sample was deposited on the bottom electrode, a thin layer of polydimethylsiloxane (PDMS) film was spin-coated on the Car_Zn·(MeCN) crystal as a protection layer. Then, another Au-coated SiO_2 substrate was firmly laminated on the PDMS layer to serve as the top electrode. Two copper wires were fixed on the Au layer to transmit electrical signals to the measuring equipment, and a PDMS film was coated on the reverse side of the top substrates to protect the device, avoiding substrate damage under a strong mechanical impact force. The device was finally packed with Kapton tape.

Characterization of the power generator. The peptide-MOF-based generator was vertically fixed on a stainless-steel plate, which was mounted on a precision linear slide stage. A linear motor (E1100-RS-HC type with Force Control, LinMot) was used to periodically press the power generator, and the pressure force was measured using a force sensor (DYZ-101, Bengbu Ocean Sensing System Engineering Co. Ltd). The outputs of the generator were recorded by a low-noise voltage preamplifier (Stanford SR560), and a constant measurement drift was subtracted from the open-circuit voltage data. The generator and measuring instruments were placed in a Faraday cage to avoid interference from the environment.

Supplementary Figures

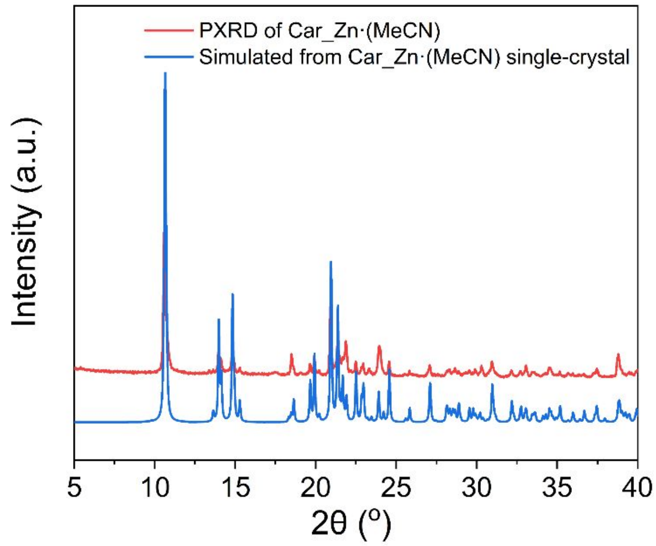

Supplementary Figure S1. PXRD spectrum of Car_Zn·(MeCN) (red) and calculated diffractogram of a Car_Zn·(MeCN) single crystal (blue).

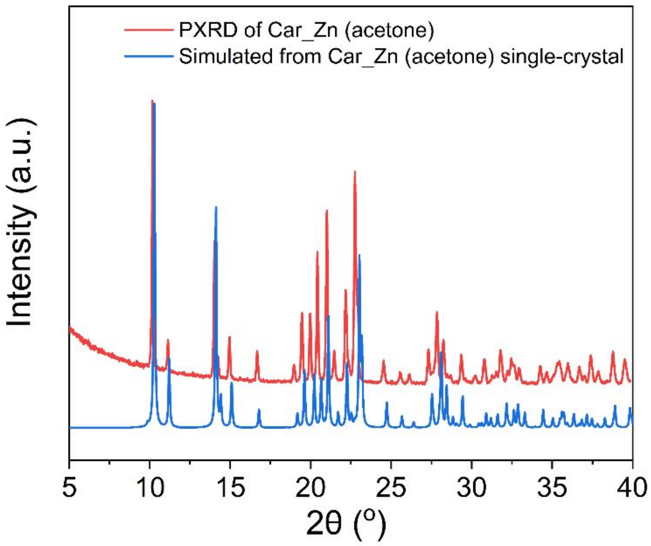

Supplementary Figure S2. PXRD spectrum of Car_Zn·(Acetone) (red) and calculated diffractogram of a Car_Zn·(Acetone) single crystal (blue).

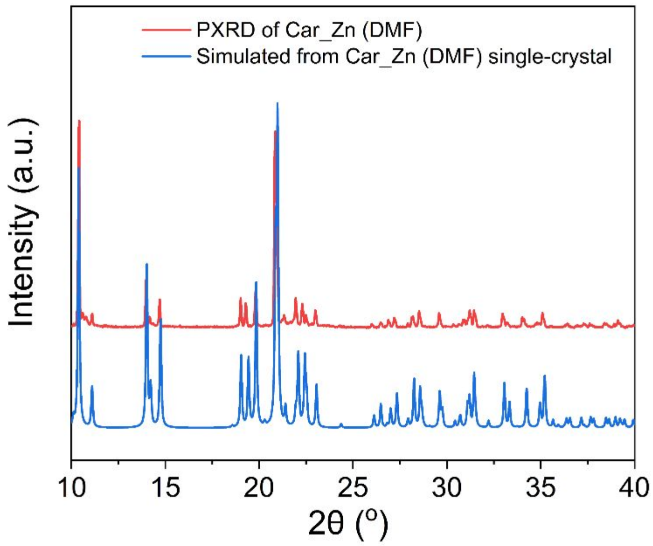

Supplementary Figure S3. PXRD spectrum of Car_Zn·(DMF) (red) and calculated diffractogram of a Car_Zn·(DMF) single crystal (blue).

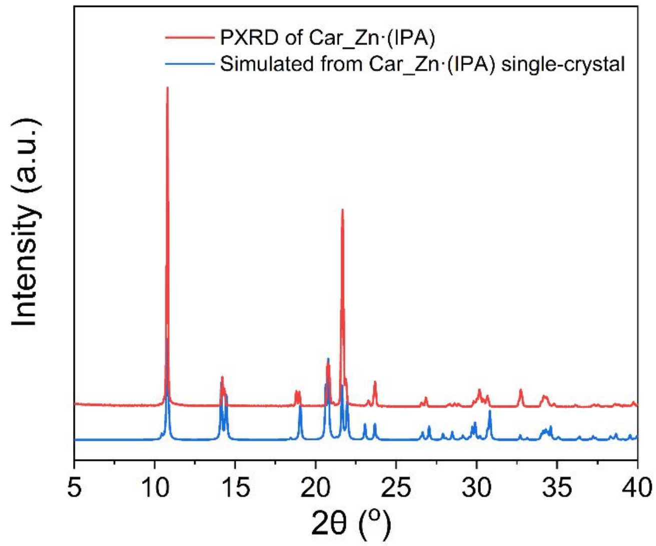

Supplementary Figure S4. PXRD spectrum of Car_Zn·(IPA) (red) and calculated diffractogram of a Car_Zn·(IPA) single crystal (blue).

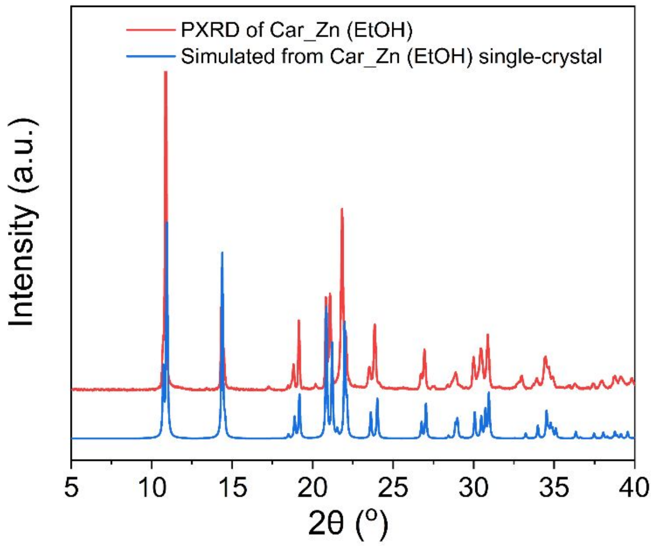

Supplementary Figure S5. PXRD spectrum of Car_Zn·(EtOH) (red) and calculated diffractogram of a Car_Zn·(EtOH) single crystal (blue).

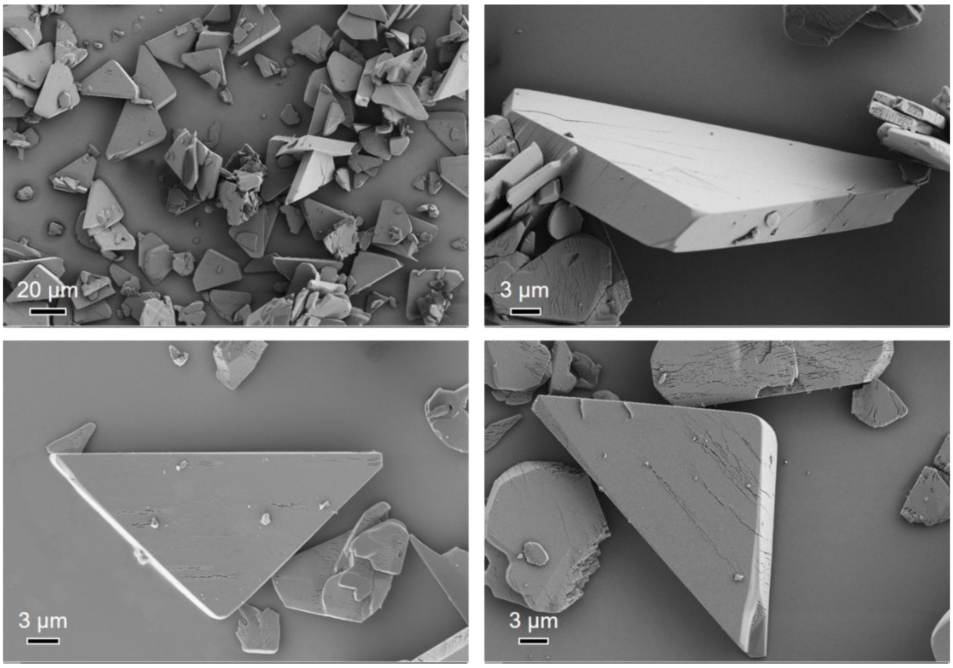

Supplementary Figure S6. SEM images showing the triangular prism morphology of Car_Zn·(IPA).

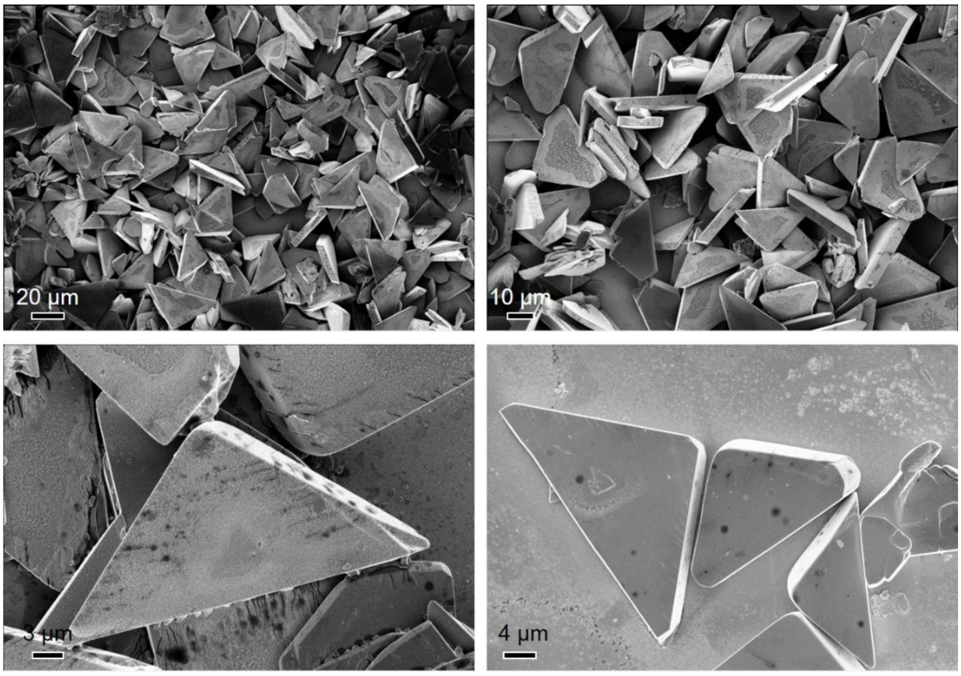

Supplementary Figure S7. SEM images showing the triangular prism morphology of Car_Zn·(EtOH).

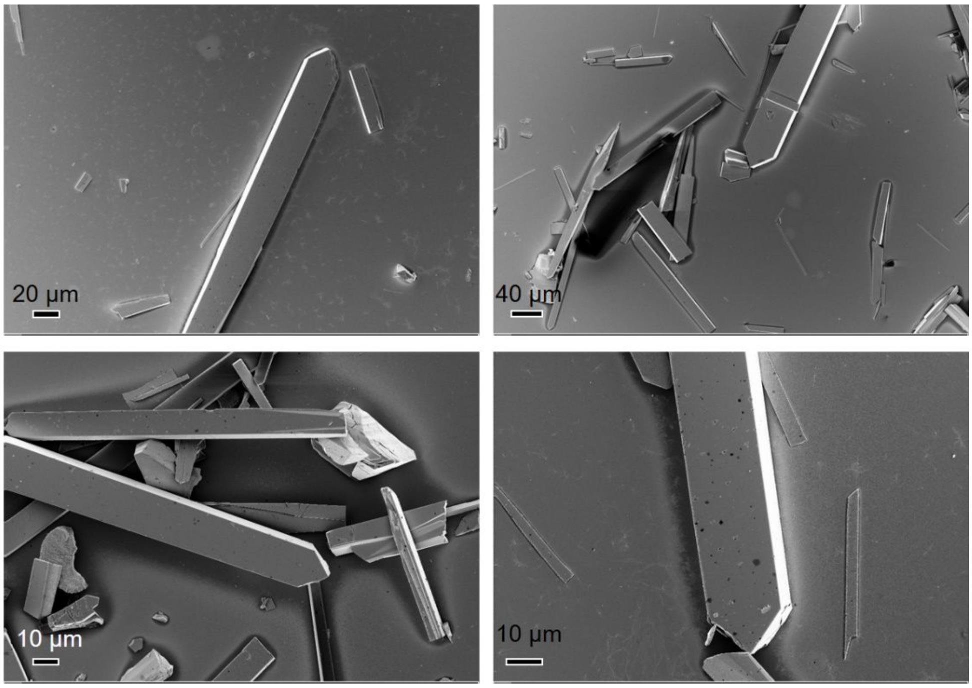

Supplementary Figure S8. SEM images showing the triangular prism morphology of Car_Zn·(DMF).

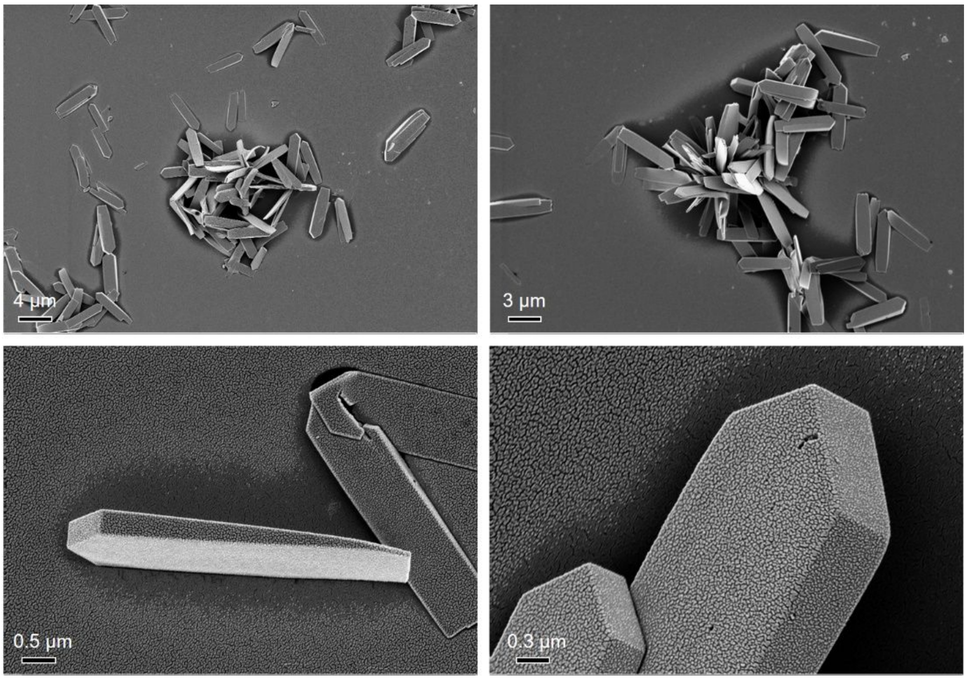

Supplementary Figure S9. SEM images showing the rectangular prism morphology of Car_Zn·(Acetone).

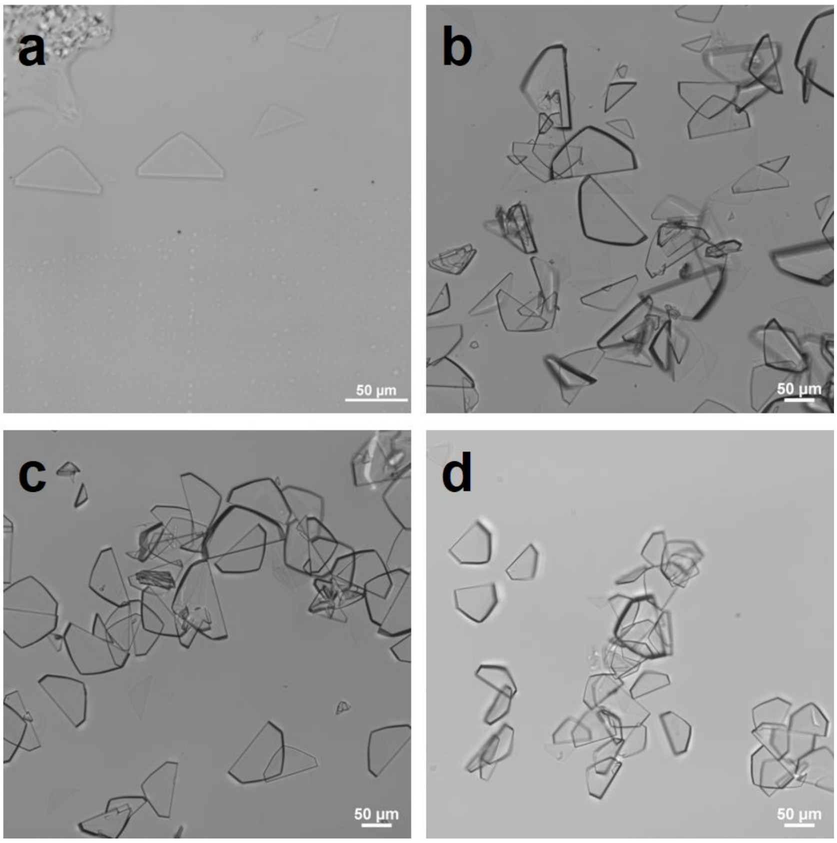

Supplementary Figure S10. Optical microscopy images of Car_Zn·(EtOH) morphology formed at different EtOH: water volume ratios: (a) 5:2, (b) 4:2, (c) 3:2, and (d) 2.5:2.

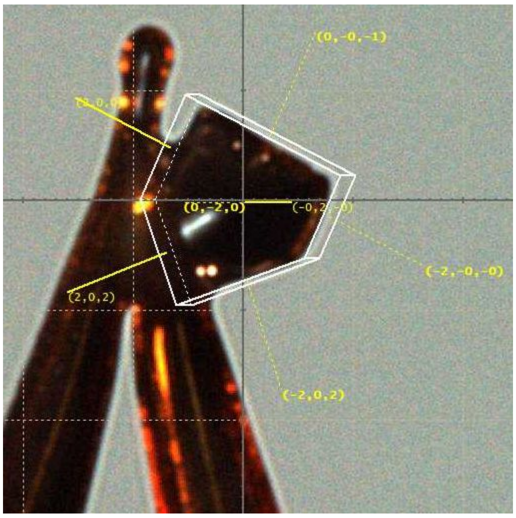

Supplementary Figure S11. Crystal face measurement of the Car_Zn·(EtOH) single crystal with respect to the rectangular prism shaped morphology. Car_Zn·(EtOH) single crystal is shown mounted on a MiTeGen loop. The crystal is highlighted in the center of the picture and the crystal faces are shown.

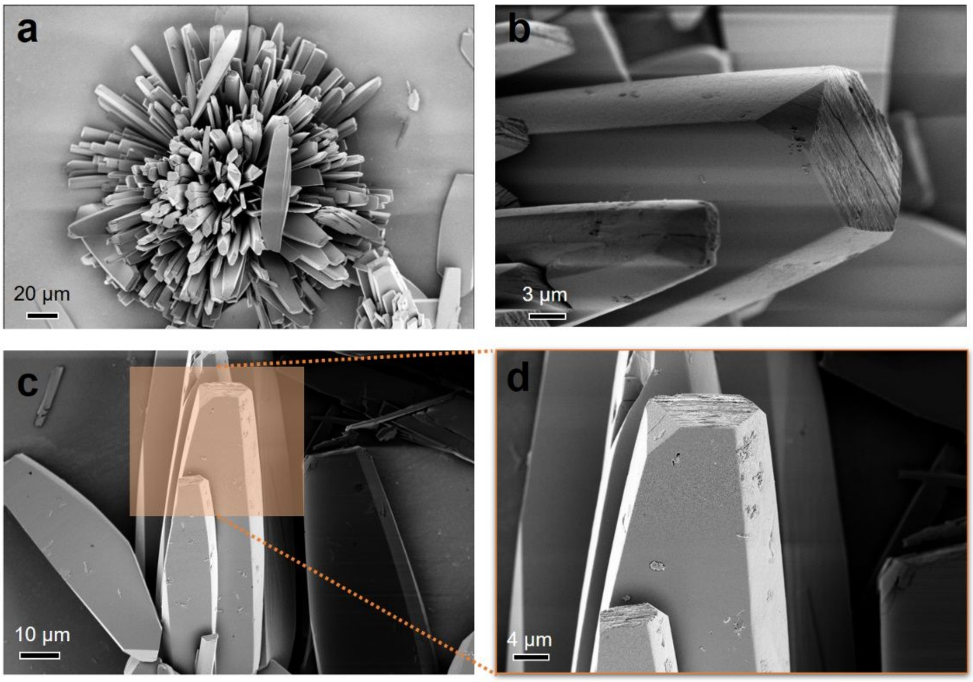

Supplementary Figure S12. (a-d) SEM images showing the hexagonal prism morphology of Car_Zn·(MeCN).

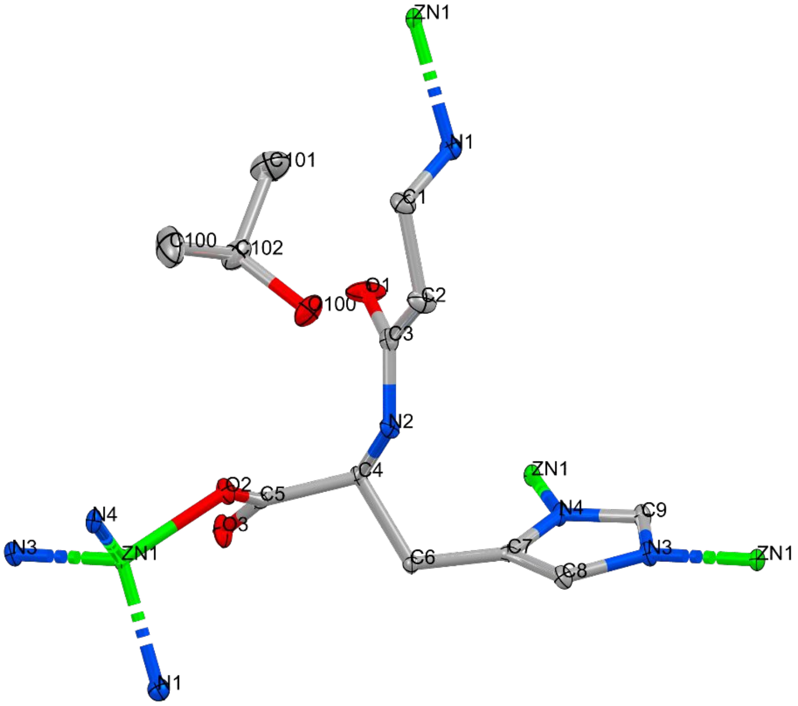

Supplementary Figure S13. ORTEP diagram of Car_Zn·(IPA) single crystal with the thermal ellipsoids set at 50% probability.

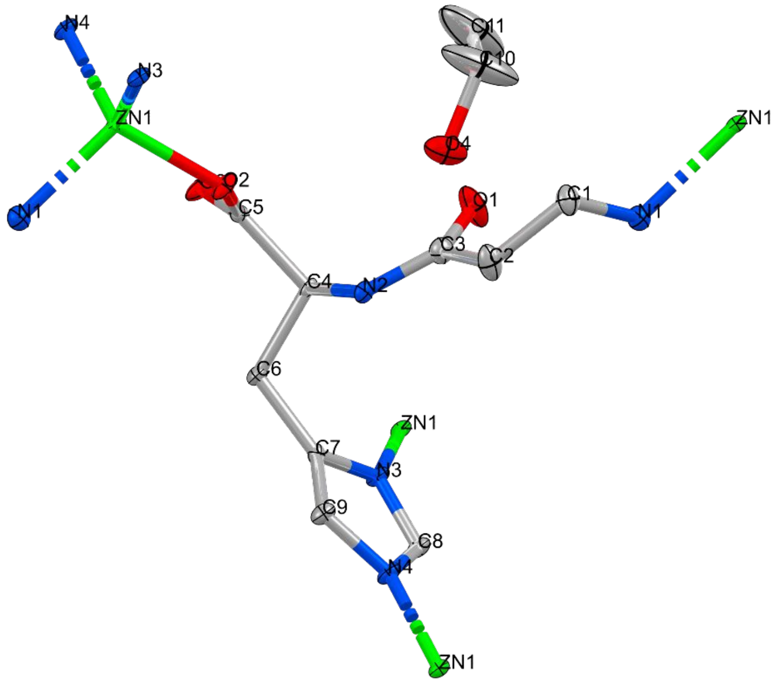

Supplementary Figure S14. ORTEP diagram of Car_Zn·(EtOH) single crystal with the thermal ellipsoids set at 50% probability.

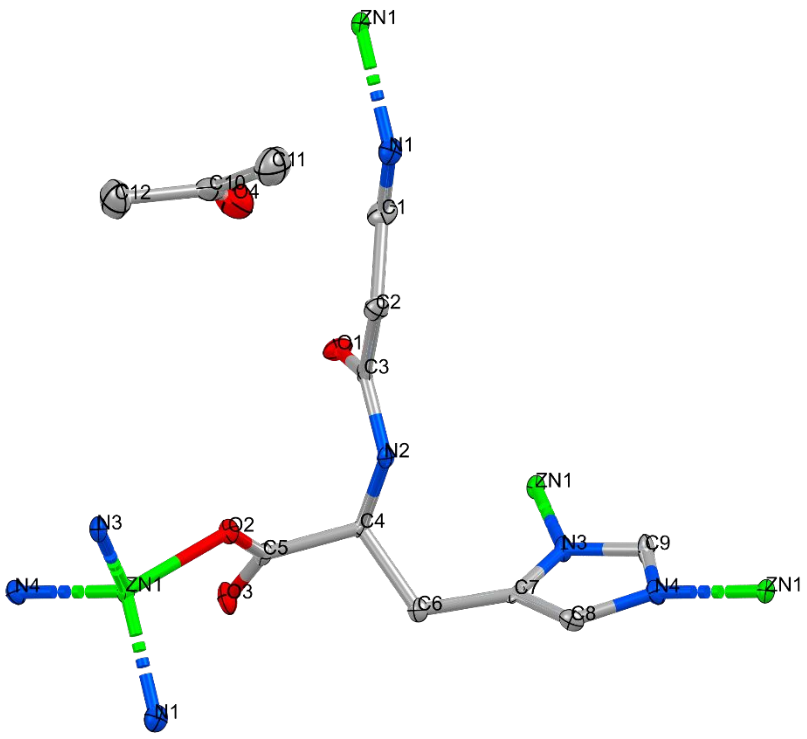

Supplementary Figure S15. ORTEP diagram of Car_Zn·(Acetone) single crystal with the thermal ellipsoids set at 50% probability.

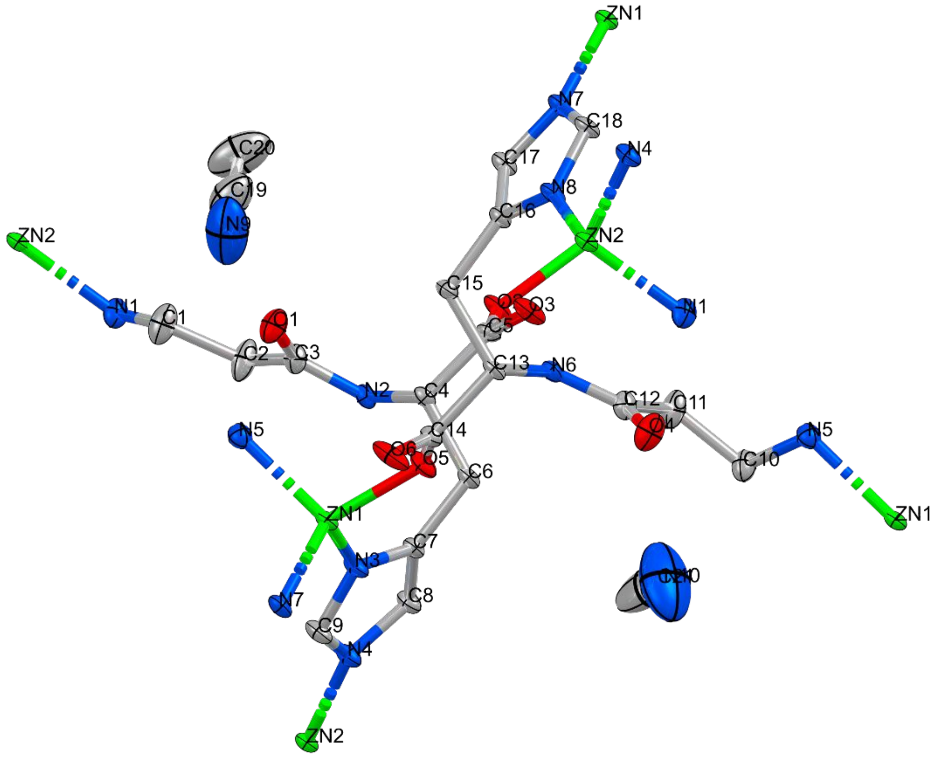

Supplementary Figure S16. ORTEP diagram of Car_Zn·(MeCN) single crystal with the thermal ellipsoids set at 50% probability.

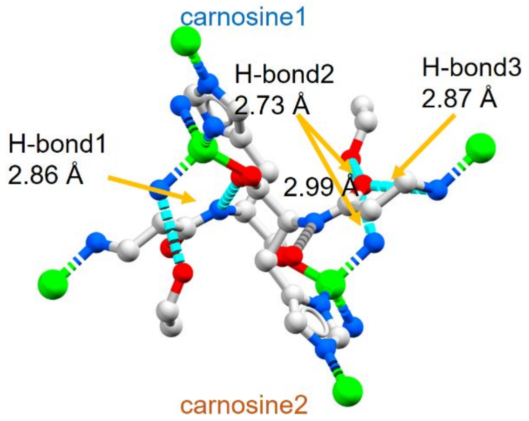

Supplementary Figure S17. The hydrogen-bonding pattern of Car_Zn·(EtOH). The guest (EtOH) molecules orient toward the amide and amine groups on the carnosine linker, forming guest-host (EtOH-Car) hydrogen bonds (O4–H4A···O1, 2.73 Å; N1–H1A···O4, 2.99 Å). The carnosine linker forms two hydrogen bonds: one is the host-host (Car-Car) intermolecular hydrogen bond (N2–H2···O3, 2.86 Å), and the other is intramolecular hydrogen bond (N1–H1A···O1, 2.87 Å).

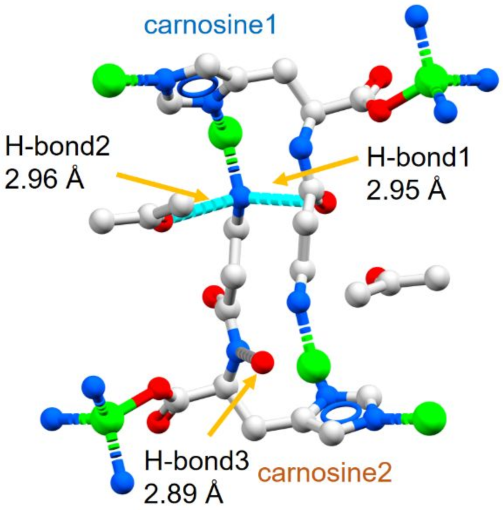

Supplementary Figure S18. The hydrogen-bonding pattern of Car_Zn·(Acetone). The guest (Acetone) molecules point toward the amine groups on the carnosine linker, forming guest-host (Acetone-Car) hydrogen bond (N1–H1D···O4, 2.96 Å). The carnosine linker forms two hydrogen bonds: one is the host-host (Car-Car) intermolecular hydrogen bond (N2–H2···O3, 2.89 Å), and the other is intermolecular hydrogen bond (N1–H1C···O1, 2.95 Å).

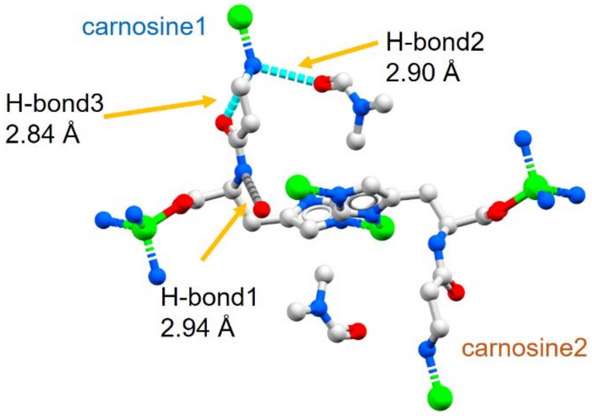

Supplementary Figure S19. The hydrogen-bonding pattern of Car_Zn·(DMF). The guest (DMF) molecules head toward the amine groups on the carnosine linker, forming guest-host (DMF-Car) hydrogen bond (N3–H3···O4, 2.90 Å). The carnosine linker forms two hydrogen bonds: one is the host-host (Car-Car) intermolecular hydrogen bond (N8–H8···O13, 2.94 Å), and the other is intramolecular hydrogen bond (N3–H3B···O5, 2.84 Å).

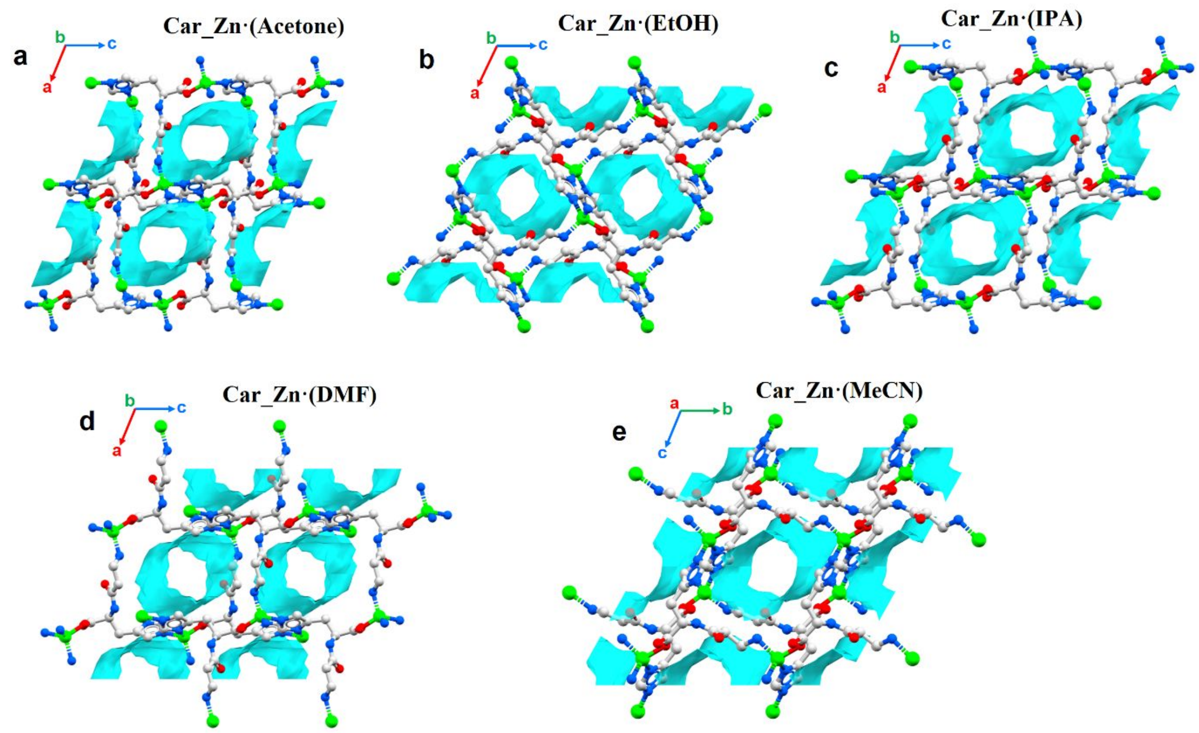

Supplementary Figure S20. (a) Car_Zn·(Acetone), (b) Car_Zn·(EtOH), (c) Car_Zn·(IPA), and (d) Car_Zn·(DMF) the voids represent the channels observed along the b axis calculated by Mercury, and (e) represent the channels of Car_Zn·(MeCN) as viewed along the a-axis.

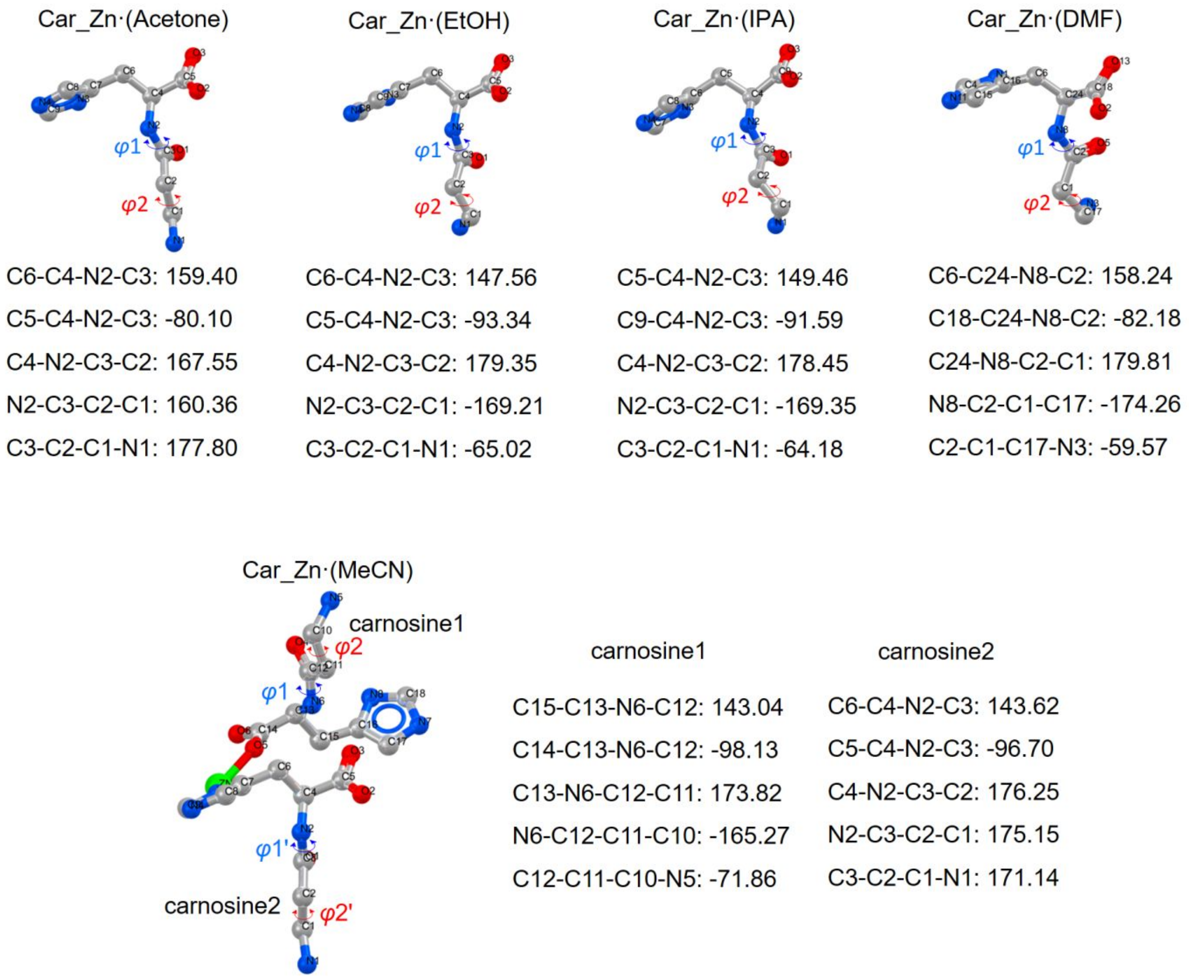

Supplementary Figure S21. Top: Torsion angles of the carnosine linker in Car_Zn·(Acetone), Car_Zn·(EtOH), Car_Zn·(IPA), and Car_Zn·(DMF). Bottom: Torsion angles of two distinct carnosine linkers in Car_Zn·(MeCN).

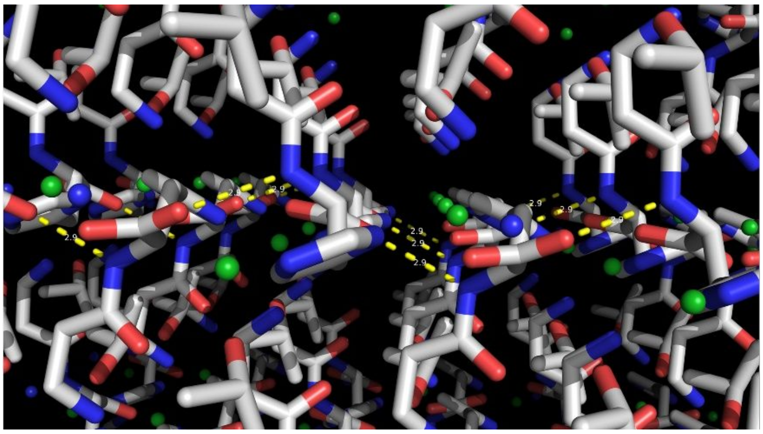

Supplementary Figure S22. Side view of the interframework hydrogen-bond pattern of Car_Zn·(IPA). The carboxylate and amide groups (N2–H20...O3, 2.89 Å) on the carnosine skeleton form intermolecular hydrogen bond interactions.

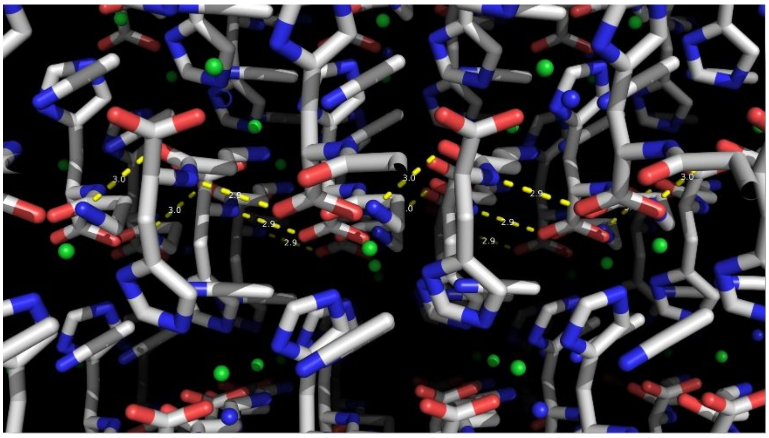

Supplementary Figure S23. Side view of the interframework hydrogen-bond pattern of Car_Zn·(MeCN). The amide groups formed an antiparallel β-sheet-like intermolecular hydrogen bond network (N2-H2...O6, 2.89 Å; N6-H6 ...O3, 2.97 Å) between the carboxylate and amine groups on the adjacent linker.

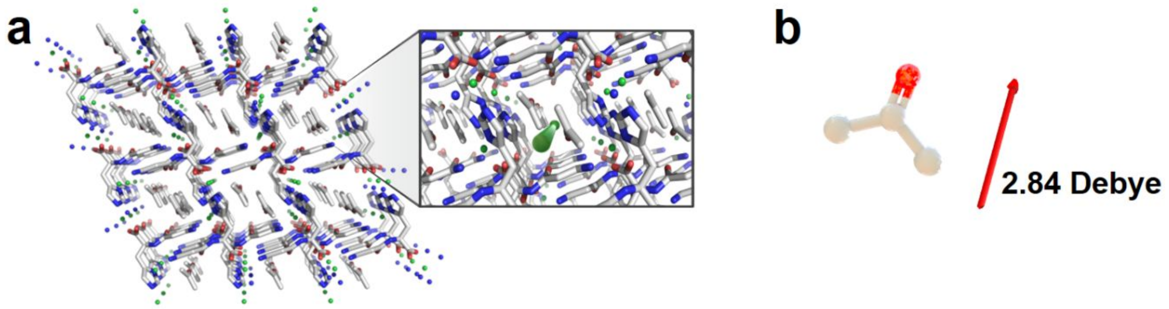

Supplementary Figure S24. Supramolecular packing pattern in Car_Zn·(Acetone) viewed down the b-axis, revealing directional guest solvent acetone molecule alignment in the Car_Zn framework channel. (B) Molecule dipole of acetone. Car_Zn·(Acetone) also demonstrates higher piezoelectric charge constants along the axis parallel to the solvent molecular dipole moment; this increased polarization is offset by the longitudinal mechanical stability to yield a modest piezoelectric response (Table S5). The acetone-mediated assembly achieves dense supramolecular packing in the pore walls along the crystallographic *a* and *c* axes (c_{11} = 23.8GPa, c_{33} = 41.2 GPa), with the hydrogen bonding network parallel to the *b* axis also yielding high c_{22} = 26.8 GPa *via* carnosine-carnosine and carnosine-acetone interactions.

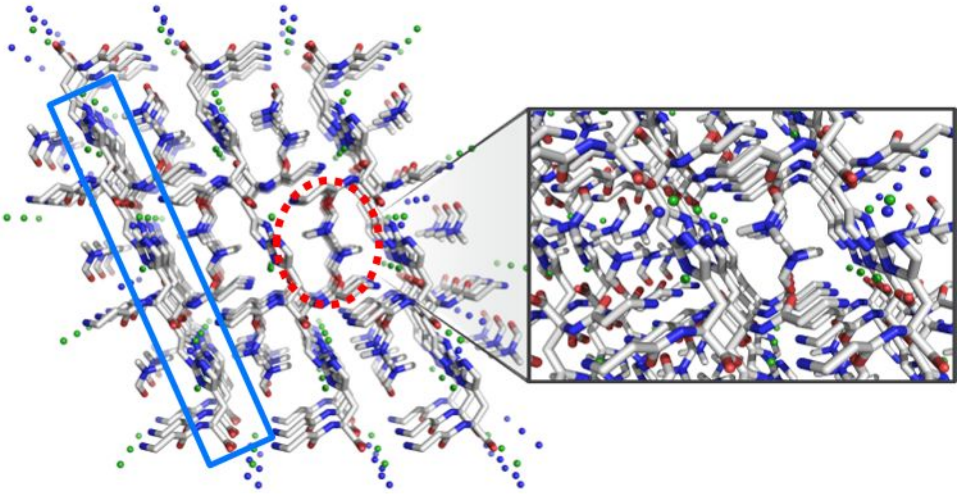

Supplementary Figure S25. Supramolecular packing pattern in Car_Zn·(DMF) viewed down the *b*-axis. In Car_Zn·(DMF), dense packing along the *c* axis is formed by chains of metal ions and carnosine molecules, resulting in a high predicted c_{33} of 47 GPa (highlighted by the blue rectangle). Along the *a* and *b* axes, the inter-ion spacing is the largest of any of the six crystals. There is large spacing between the solvent molecules (examples circled in red) and the carnosine, with limited intermolecular interaction.

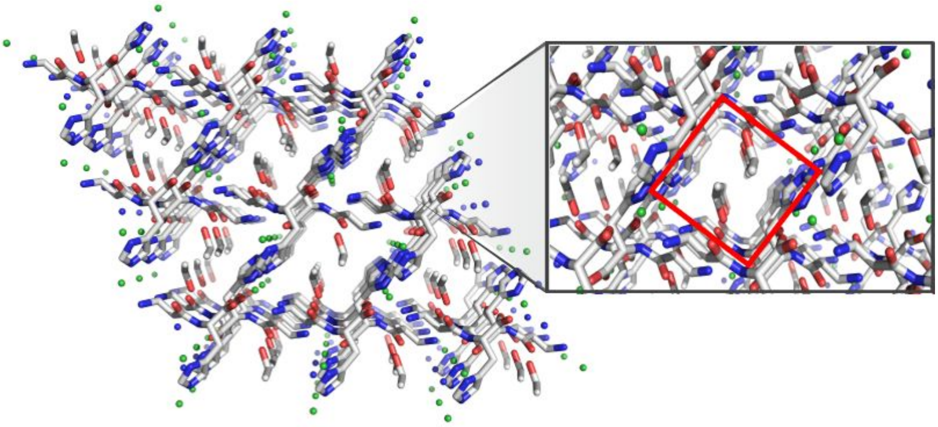

Supplementary Figure S26. Supramolecular packing pattern in Car_Zn·(EtOH) viewed down the b-axis. Car_Zn·(EtOH) demonstrates unique mechanical properties due to its diamond-shaped pores that significantly increase the shear stiffness (c_{55} = 15.6 GPa, Table S6) compared to the other crystals. The relatively low charge tensor values may be due to the diametrically-opposed orientations of the two alcohol molecules in each pore. As a result, the ethanol molecular dipoles cancel out and do not contribute to the piezoelectric response.

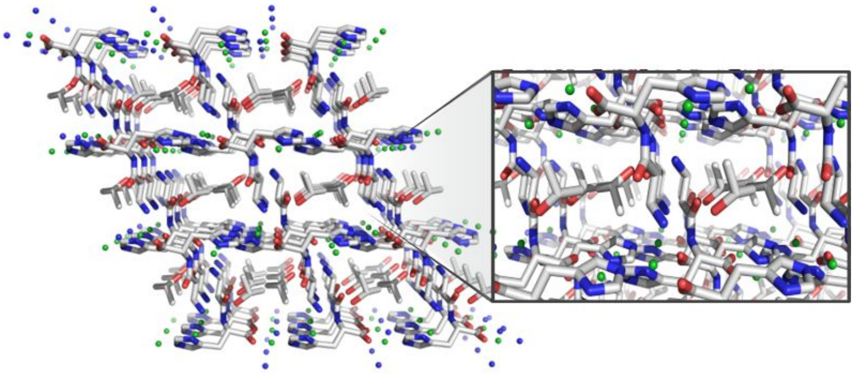

Supplementary Figure S27. Supramolecular packing pattern in Car_Zn·(IPA) viewed down the b-axis. In Car_Zn·(IPA), the pores are off-center near the corners of the cell. Therefore, when a shearing force is applied along the c axis, the molecular layers are more readily pushed close together (c_{66} = 2.8 GPa, the lowest stiffness constant of all the crystals), facilitating a maximal piezoelectric response of d_{36} = 7.3 pC/N (Table S7).

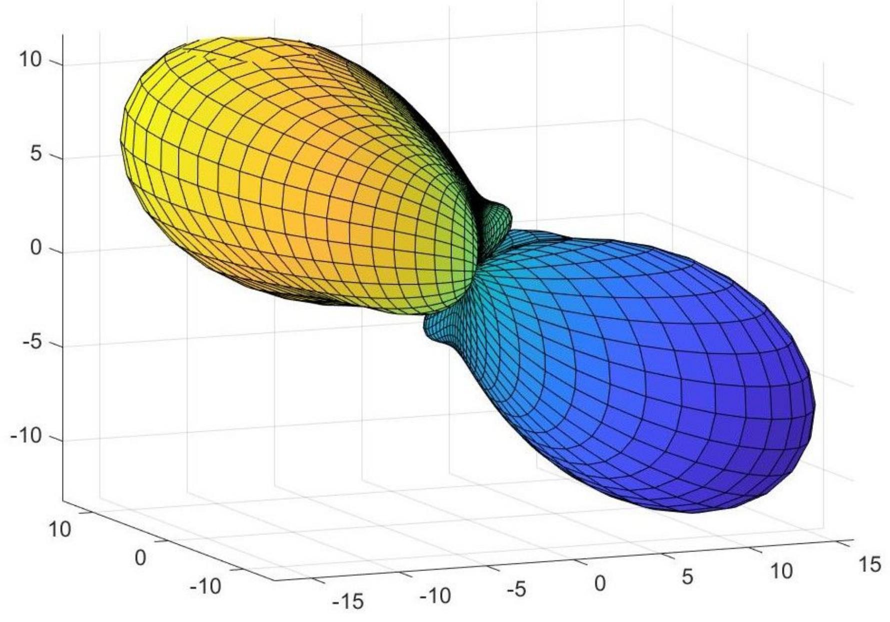

Supplementary Figure S28. 3D surface plot of computed piezoelectric coefficients of Car_Zn·(MeCN) single crystal.

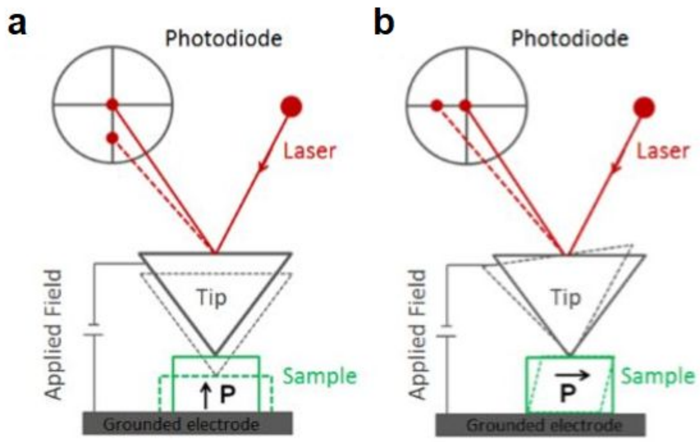

Supplementary Figure S29. Schematics of PFM operation. A sample deforms in response to an applied AC voltage, which causes the cantilever to deflect. (a) When the polarization is parallel to the applied voltage, the sample will generate an out-of-plane piezoresponse, measured by the tip-photodiode system as a vertical deflection. (b) When the polarisation is perpendicular to the applied voltage, the sample will generate an in-plane piezoresponse that is measured as a lateral deflection by the tip-photodiode system.

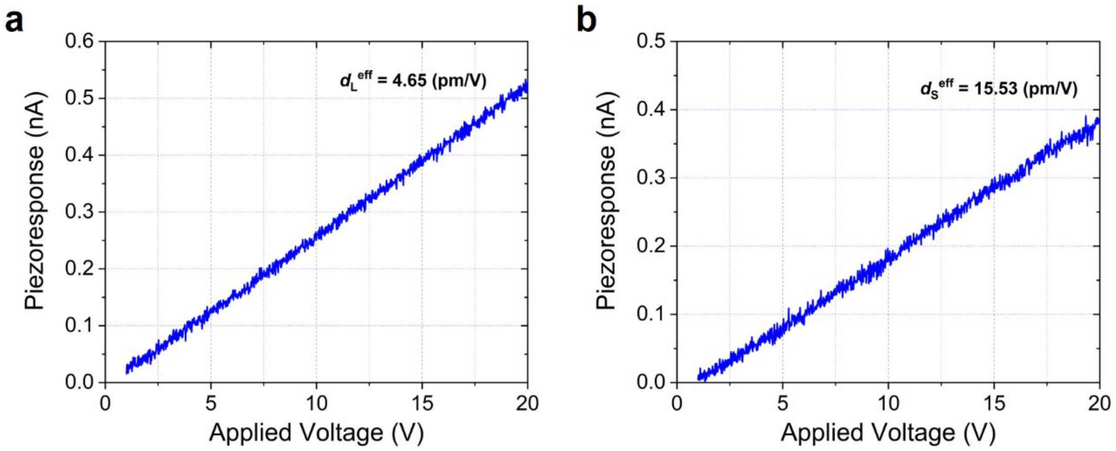

Supplementary Figure S30. Linear relationship between the (a) longitudinal and (b) shear piezoresponse of the Car_Zn·(MeCN) crystal as measured by the photodiode system and under applied voltage.

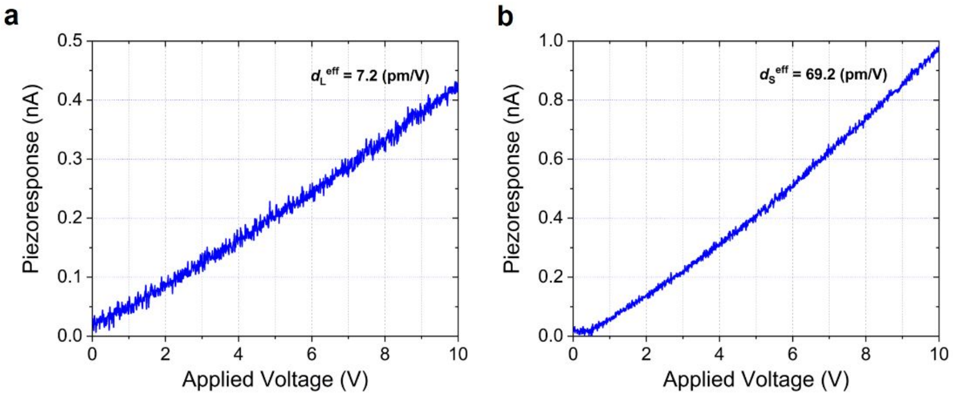

Supplementary Figure S31. Two commercially available lithium niobate samples were used as positive controls to quantify the accuracy of the PFM system for longitudinal and shear piezoelectric coefficient measurements. Linear relationship between the (a) longitudinal piezoresponse of periodically poled lithium niobate (NT-MDT, Russia) and (b) shear piezoresponse of Y-cut LN (Siegert Wafer, Germany) as measured by the photodiode system and applied voltage. The measured longitudinal piezoresponse coefficient of Z-cut face of lithium niobate is 7.2 pm/V, in perfect agreement with a previous report[18–20]. In the case of Y-cut LN, a non-zero $d_{24} = d_{15}$ shear coefficient can be detected, which is generally considered to be in the range of 68-70 pm/V. The above results show that our PFM measurement method can accurately provide the effective piezoelectric coefficient of a peptide single crystal.

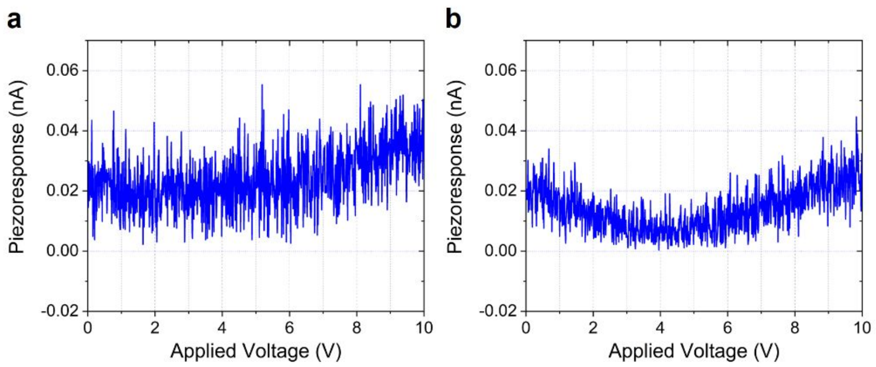

Supplementary Figure S32. Negative control for the PFM measurement. Miniscule, poorly linear relationship between the (a) longitudinal piezoresponse and (b) shear piezoresponse of amorphous glass as measured by the photodiode system under applied voltage.

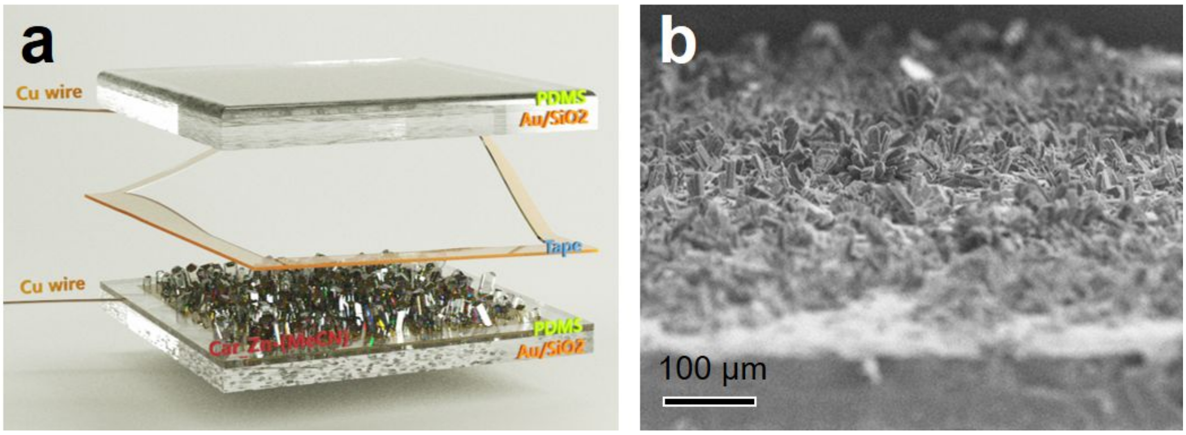

Supplementary Figure S33. (a) Schematic of the proof-of-concept peptide-MOF based energy harvesting device. The piezoelectric energy harvesting device was designed and fabricated by sandwiching the Car_Zn·(MeCN) crystals array between two gold-coated silicon dioxide substrates. (b) SEM image of Car_Zn·(MeCN) crystals array.

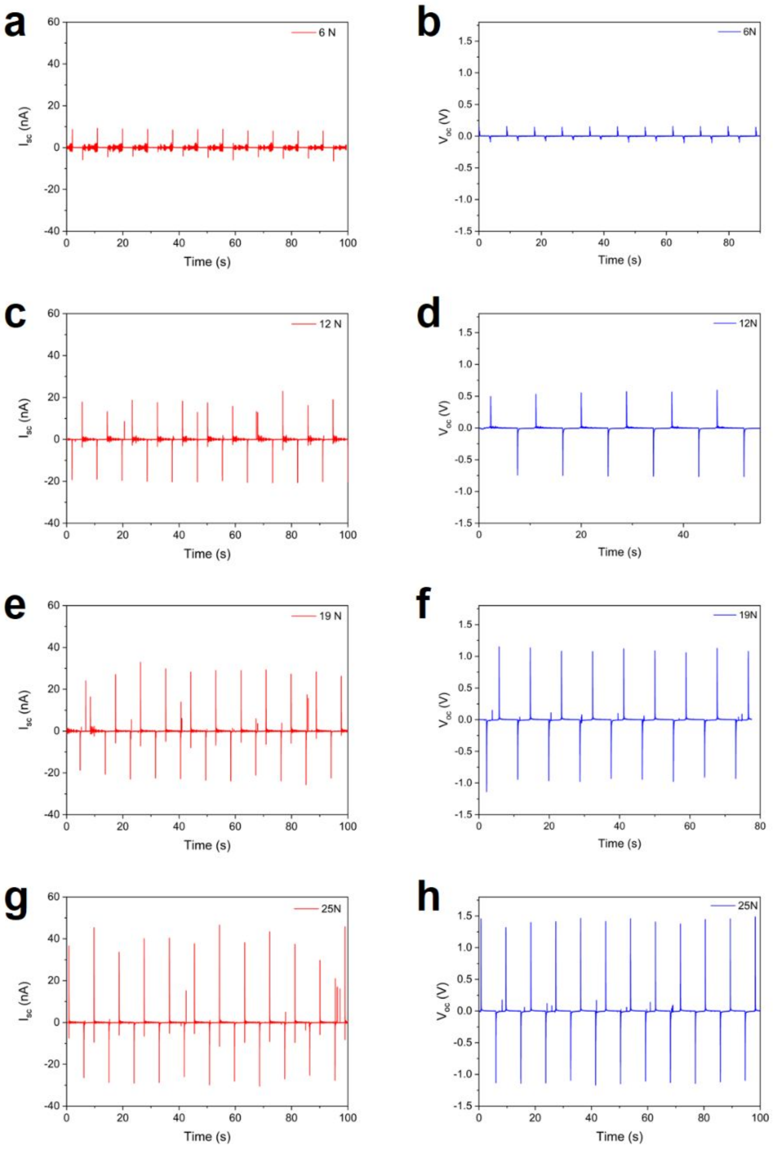

Supplementary Figure S34. Characterization of Car_Zn·(MeCN)-based piezoelectric energy generator. (a, c, e, g) Open circuit voltage and (b, d, f, h) short-circuit current of piezoelectric harvester upon applied force = of (a, b) 6, (c, d) 12, (e, f) 19, and (g, h) 25 N.

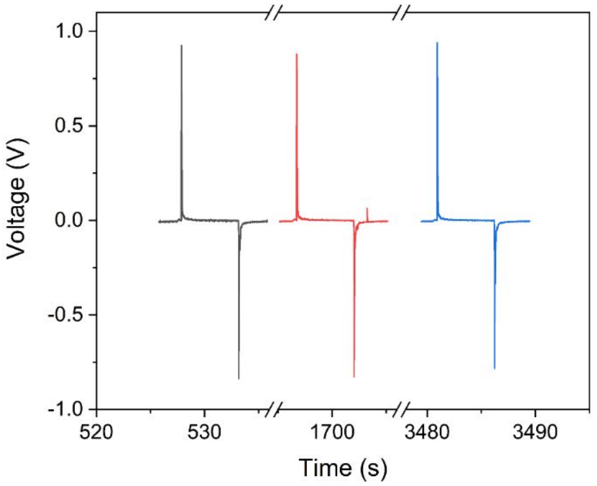

Supplementary Figure S35. Enlarged open circuit voltage waveform from the stability measurement of Car_Zn·(MeCN)-based piezoelectric energy generator (Figure 4F in the main text) at different time points (530 s, 1700 s, and 3480 s), showing the sustained large, unvarying voltage output.

Supplementary Tables

Supplementary Table 1. Data collection and refinement statistics of Car_Zn single crystals.

Crystal data	Car_Zn·(MeCN)	Car_Zn·(IPA)	Car_Zn·(EtOH)	Car_Zn·(Acetone)
CCDC Deposition Number	2104697	2104698	2104699	2104700
Chemical formula	C22 H30 N10 O6 Zn2	C12 H20 N4 O4 Zn	C11 H18 N4 O4 Zn	C12 H18 N4 O4 Zn
Mr	661.34	349.69	335.66	347.69
Crystal system	Triclinic	Monoclinic	Monoclinic	Monoclinic
Space group	*P*1	*P* 21	*P* 21	*P* 21
Hall group	*P*1	*P* 2yb	*P* 2yb	*P* 2yb
a (Å)	9.0839(1)	9.3164(8)	9.3937(3)	9.3008(8)
b (Å)	9.5608(2)	9.2981(8)	9.2495(3)	8.7747(7)
c (Å)	9.5994(1)	9.6214(8)	9.3999(3)	9.7001(7)
α (°)	119.009(2)	90	90	90
β (°)	90.208(1)	118.356(2)	118.634(2)	112.461(3)
γ (°)	95.611(1)	90	90	90
V ($Å_3$)	724.37(2)	733.45(11)	716.84(4)	731.59(10)
Z , Z'	1	2	2	2
μ (mm^{-1})	1.709	1.696	1.732	1.700
Temperature (K)	100 K	100 K	100 K	100 K
Data collection				
Diffractometer	Rigaku XtaLab^{Pro} MoK α radiation (λ=0.71073Å)	Bruker Kappa ApexII with MoK α radiation (λ=0.71073Å)	Bruker Kappa ApexII with MoK α radiation (λ=0.71073Å)	Bruker Kappa ApexII with MoK α radiation (λ=0.71073Å)
Absorption correction	multi-scan	multi-scan	multi-scan	multi-scan
No. reflections (unique)	30580(19782)	14620(4317)	7150(4739)	8671(3098)
Rint	0.0457	0.0428	0.0588	0.0688
Refinement				
R [*F*2 > 2σ(*F*2)]	0.0499	0.0289	0.0282	0.0331
wR(*F*2)	0.1453	0.0482	0.0602	0.0541
Goodness-of-fit	1.045	0.985	1.000	0.976
No. of data/restraints/parameters	19782/3/364	4317/1/193	4739/1/184	3098/1/192
H-atom treatment	H-atom parameters constrained	H-atom parameters constrained	H-atom parameters constrained	H-atom parameters constrained

Supplementary Table 2. Computed elastic constants of Car_Zn MOF single crystals. All values are in GPa. c_{11}, c_{22}, and c_{33} are the longitudinal stiffness constants; c_{44}, c_{55} and c_{66} are the shear stiffness constants.

**Elastic Stiffness**	Car_Zn·(MeCN)	Car_Zn·(IPA)	Car_Zn·(Acetone)	Car_Zn·(DMF)	Car_Zn·(EtOH)
c_{11}	29.9	23.0	23.9	29.2	22.0
c_{22}	19.2	25.3	26.8	29.0	30.0
c_{33}	30.6	36.5	41.2	47.2	32.3
c_{44}	10.8	5.1	10.6	9.8	10.0
c_{55}	8.4	8.6	10.3	10.7	15.6
c_{66}	5.2	2.8	5.7	5.7	7.1

Supplementary Table 3. Young's modulus values approximated using DFT-calculated elastic stiffness and compliance tensors. All values are in GPa.

Crystal Solvent	Nye	Voigt-Reuss-Hill	Average
Car_Zn·(MeCN)	3.8/5.7	12.9	7.5
Car_Zn·(IPA)	9.9/7.6	8.2	8.6
Car_Zn·(Acetone)	11.7/13.0	16.5	14.1
Car_Zn·(DMF)	14.4/16.8	18.2	16.5
Car_Zn·(EtOH)	34.1/21.5	18.8	26.5

More details on the approximations used to derive Young's moduli from the stiffness matrix component are provided in ref [21].

Supplementary Table 4. Calculated piezoelectric charge tensor components e_{ij} (in units C/m^2), strain tensor components d_{ik} (pm/V), and voltage tensor components g_{ij} (mV m/N), of Car_Zn·(MeCN) single crystal.

Charge Tensor (C/m^2)

$$\begin{pmatrix} -0.32 & -0.31 & -0.33 & -0.03 & 0.15 & 0.01 \\ 0.05 & 0.08 & 0.05 & 0.00 & 0.02 & -0.03 \\ 0.11 & 0.19 & 0.14 & 0.03 & 0.04 & -0.05 \end{pmatrix}$$

Strain Tensor (pm/V)

$$\begin{pmatrix} -10.7 & -16.3 & -10.9 & -2.7 & 18.2 & 2.0 \\ 1.8 & 4.2 & 1.7 & 0.1 & 2.4 & -6.3 \\ 3.8 & 9.8 & 4.7 & 2.4 & 4.3 & -9.9 \end{pmatrix}$$

Voltage Tensor (mV m/N)

$$\begin{pmatrix} -257 & -390 & -261 & -65 & 435 & 48 \\ 52 & 122 & 50 & 2 & 70 & -186 \\ 103 & 265 & 126 & 65 & 117 & -268 \end{pmatrix}$$

Supplementary Table 5. Calculated piezoelectric charge tensor components e_{ij} (C/m^2), strain tensor components d_{ik} (pm/V) and voltage tensor components g_{ij} (mV m/N), of Car_Zn·(DMF).

Charge Tensor (C/m^2)

$$\begin{pmatrix} 0.00 & 0.00 & 0.00 & -0.04 & 0.00 & 0.04 \\ 0.04 & 0.08 & 0.08 & 0.00 & 0.00 & 0.00 \\ 0.00 & 0.00 & 0.00 & 0.02 & 0.00 & 0.01 \end{pmatrix}$$

Strain Tensor (pm/V)

$$\begin{pmatrix} 0.0 & 0.0 & 0.0 & -3.7 & 0.0 & 6.9 \\ 1.3 & 2.7 & 1.6 & 0.0 & 0.4 & 0.0 \\ 0.0 & 0.0 & 0.0 & 2.0 & 0.0 & 2.5 \end{pmatrix}$$

Voltage Tensor (mV m/N)

$$\begin{pmatrix} 0 & 0 & 0 & -93 & 0 & 177 \\ 41 & 83 & 51 & 0 & 12 & 0 \\ 0 & 0 & 0 & 65 & 0 & 83 \end{pmatrix}$$

Supplementary Table 6. Calculated piezoelectric charge tensor components e_{ij} (C/m^2), strain tensor components d_{ik} (pm/V) and voltage tensor components g_{ij} (mV m/N), of Car_Zn·(Acetone).

Charge Tensor (C/m^2)

$$\begin{pmatrix} 0.00 & 0.00 & 0.00 & -0.007 & 0.00 & 0.06 \\ 0.10 & 0.13 & 0.15 & 0.00 & -0.04 & 0.00 \\ 0.00 & 0.00 & 0.00 & 0.05 & 0.00 & 0.02 \end{pmatrix}$$

Strain Tensor (pm/V)

$$\begin{pmatrix} 0.0 & 0.0 & 0.0 & -0.7 & 0.0 & 11.2 \\ 4.0 & 4.8 & 3.5 & 0.0 & -3.6 & 0.0 \\ 0.0 & 0.0 & 0.0 & 4.6 & 0.0 & 2.8 \end{pmatrix}$$

Voltage Tensor (mV m/N)

$$\begin{pmatrix} 0 & 0 & 0 & -24 & 0 & 386 \\ 116 & 139 & 103 & 0 & -105 & 0 \\ 0 & 0 & 0 & 148 & 0 & 91 \end{pmatrix}$$

Supplementary Table 7. Calculated piezoelectric charge tensor components e_{ij} (C/m^2), strain tensor components d_{ik} (pm/V), and voltage tensor components g_{ij} (mV m/N), of Car_Zn·(EtOH).

Charge Tensor (C/m^2)

$$\begin{pmatrix} 0.00 & 0.00 & 0.00 & -0.009 & 0.00 & 0.04 \\ -0.02 & 0.05 & 0.04 & 0.00 & 0.00 & 0.00 \\ 0.00 & 0.00 & 0.00 & -0.02 & 0.00 & 0.00 \end{pmatrix}$$

Strain Tensor (pm/V)

$$\begin{pmatrix} 0.0 & 0.0 & 0.0 & -0.09 & 0.0 & 5.5 \\ -0.9 & 1.8 & 1.2 & 0.0 & 0.2 & 0.0 \\ 0.0 & 0.0 & 0.0 & -1.5 & 0.0 & -0.6 \end{pmatrix}$$

Voltage Tensor (mV m/N)

$$\begin{pmatrix} 0.0 & 0.0 & 0.0 & -30 & 0.0 & 190 \\ -27 & 54 & 38 & 0.0 & 5 & 0.0 \\ 0.0 & 0.0 & 0.0 & -50 & 0.0 & -21 \end{pmatrix}$$

Supplementary Table 8. Calculated piezoelectric charge tensor components e_{ij} (C/m^2), strain tensor components d_{ik} (pm/V), and voltage tensor components g_{ij} (mV m/N), of Car_Zn·(IPA).

Charge Tensor (C/m^2)

$$\begin{pmatrix} 0.00 & 0.00 & 0.00 & 0.03 & 0.00 & 0.02 \\ 0.03 & 0.09 & -0.01 & 0.00 & -0.02 & 0.00 \\ 0.00 & 0.00 & 0.00 & 0.01 & 0.00 & 0.02 \end{pmatrix}$$

Strain Tensor (pm/V)

$$\begin{pmatrix} 0.0 & 0.0 & 0.0 & 5.2 & 0.0 & 5.6 \\ 1.3 & 3.5 & -0.2 & 0.0 & -2.1 & 0.0 \\ 0.0 & 0.0 & 0.0 & 1.4 & 0.0 & 7.3 \end{pmatrix}$$

Voltage Tensor (mV m/N)

$$\begin{pmatrix} 0 & 0 & 0 & 166 & 0 & 179 \\ 40 & 105 & -6 & 0 & -64 & 0 \\ 0 & 0 & 0 & 46 & 0 & 245 \end{pmatrix}$$

Supplementary Table 9. Computed dielectric constants and crystal unit cell dipole moments of Car_Zn MOF single crystals.

Crystal	Dielectric Constant	Crystal Dipole (Debye)
Car_Zn·(MeCN)	4.2	11.31 (along *a*-axis)
Car_Zn·(DMF)	3.8	3.83 (along *b*-axis)
Car_Zn·(Acetone)	3.6	13.89 (along *b*-axis)
Car_Zn·(EtOH)	3.5	1.65 (along *b*-axis)
Car_Zn·(IPA)	3.5	1.61 (along *b*-axis)

Reference:

(1) Katsoulidis, A. P.; Park, K. S.; Antypov, D.; Martí-Gastaldo, C.; Miller, G. J.; Warren, J. E.; Robertson, C. M.; Blanc, F.; Darling, G. R.; Berry, N. G.; Purton, J. A.; Adams, D. J.; Rosseinsky, M. J. Guest-Adaptable and Water-Stable Peptide-Based Porous Materials by Imidazolate Side Chain Control. *Angew. Chem. Int. Ed.* **2014**, *53* (1), 193–198.

(2) Argaman, N.; Makov, G. Density Functional Theory: An Introduction. *Am. J. Phys.* **2000**, *68* (1), 69–79.

(3) Hafner, J. Materials Simulations Using VASP—a Quantum Perspective to Materials Science. *Comput. Phys. Commun.* **2007**, *177* (1), 6–13.

(4) Perdew, J. P.; Chevary, J. A.; Vosko, S. H.; Jackson, K. A.; Pederson, M. R.; Singh, D. J.; Fiolhais, C. Atoms, Molecules, Solids, and Surfaces: Applications of the Generalized Gradient Approximation for Exchange and Correlation. *Phys. Rev. B* **1992**, *46* (11), 6671–6687.

(5) Grimme, S.; Antony, J.; Ehrlich, S.; Krieg, H. A Consistent and Accurate *Ab Initio* Parametrization of Density Functional Dispersion Correction (DFT-D) for the 94 Elements H-Pu. *J. Chem. Phys.* **2010**, *132* (15), 154104.

(6) Kresse, G.; Joubert, D. From Ultrasoft Pseudopotentials to the Projector Augmented-Wave Method. *Phys. Rev. B* **1999**, *59* (3), 1758–1775.

(7) Wu, X.; Vanderbilt, D.; Hamann, D. R. Systematic Treatment of Displacements, Strains, and Electric Fields in Density-Functional Perturbation Theory. *Phys. Rev. B* **2005**, *72* (3), 035105.

(8) Nye, J. F. *Physical Properties of Crystals: Their Representation by Tensors and Matrices*; Oxford science publications; Clarendon Press, 1985.

(9) Chung, D. H.; Buessem, W. R. The Voigt-Reuss-Hill Approximation and Elastic Moduli of Polycrystalline MgO, CaF_2, B-ZnS, ZnSe, and CdTe. *J. Appl. Phys.* **1967**, *38* (6), 2535–2540.

(10) Zuo, L.; Humbert, M.; Esling, C. Elastic Properties of Polycrystals in the Voigt-Reuss-Hill Approximation. *J. Appl. Crystallogr.* **1992**, *25* (6), 751–755.

(11) Guerin, S.; Stapleton, A.; Chovan, D.; Mouras, R.; Gleeson, M.; McKeown, C.; Noor, M. R.; Silien, C.; Rhen, F. M. F.; Kholkin, A. L.; Liu, N.; Soulimane, T.; Tofail, S. A. M.; Thompson, D. Control of Piezoelectricity in Amino Acids by Supramolecular Packing. *Nat. Mater.* **2018**, *17* (2), 180–186.

(12) Guerin, S.; Tofail, S. A. M.; Thompson, D. Longitudinal Piezoelectricity in Natural Calcite Materials: Preliminary Studies. *IEEE Trans. Dielectr. Electr. Insul.* **2018**, *25* (3), 803–807.

(13) Azuri, I.; Meirzadeh, E.; Ehre, D.; Cohen, S. R.; Rappe, A. M.; Lahav, M.; Lubomirsky, I.; Kronik, L. Unusually Large Young's Moduli of Amino Acid Molecular Crystals. *Angew. Chem. Int. Ed.* **2015**, *54* (46), 13566–13570.

(14) Momma, K.; Izumi, F. *VESTA 3* for Three-Dimensional Visualization of Crystal, Volumetric and Morphology Data. *J. Appl. Crystallogr.* **2011**, *44* (6), 1272–1276.

(15) O'Donnell, J.; Guerin, S.; Makam, P.; Cazade, P.-A.; Haq, E. U.; Tao, K.; Gazit, E.; Silien, C.; Soulimane, T.; Thompson, D.; Tofail, S. A. M. Atomistic-Benchmarking towards a Protocol Development for Rapid Quantitative Metrology of Piezoelectric Biomolecular Materials. *Appl. Mater. Today* **2020**, *21*, 100818.

(16) O'Donnell, J.; Haq, E. U.; Silien, C.; Soulimane, T.; Thompson, D.; Tofail, S. A. M. A Practical Approach for Standardization of Converse Piezoelectric Constants Obtained from Piezoresponse Force Microscopy. *J. Appl. Phys.* **2021**, *129* (18), 185104.

(17) O'Donnell, J.; Cazade, P.; Guerin, S.; Djeghader, A.; Haq, E. U.; Tao, K.; Gazit, E.; Fukada, E.; Silien, C.; Soulimane, T.; Thompson, D.; Tofail, S. A. M. Piezoelectricity of the Transmembrane Protein Ba3 Cytochrome c Oxidase. *Adv. Funct. Mater.* **2021**, *31* (28), 2100884.

(18) Smith, R. T.; Welsh, F. S. Temperature Dependence of the Elastic, Piezoelectric, and Dielectric Constants of Lithium Tantalate and Lithium Niobate. *J. Appl. Phys.* **1971**, *42* (6), 2219–2230.

(19) Warner, A. W.; Onoe, M.; Coquin, G. A. Determination of Elastic and Piezoelectric Constants for Crystals in Class (3m). *J. Acoust. Soc. Am.* **1967**, *42* (6), 1223–1231.
(20) Yue, W.; Yi-jian, J. Crystal Orientation Dependence of Piezoelectric Properties in $LiNbO_3$ and $LiTaO_3$. *Opt. Mater.* **2003**, *23* (1), 403–408.
(21) Kiely, E.; Zwane, R.; Fox, R.; Reilly, A. M.; Guerin, S. Density Functional Theory Predictions of the Mechanical Properties of Crystalline Materials. *CrystEngComm* **2021**, *23* (34), 5697–5710.
